# Multifunctional thermo-sensitive hydrogel for modulating the microenvironment in Osteoarthritis by polarizing macrophages and scavenging RONS

**DOI:** 10.1186/s12951-022-01422-9

**Published:** 2022-05-07

**Authors:** Chunrong Zhu, Shangcong Han, Xianhu Zeng, Chunxiao Zhu, Yuji Pu, Yong Sun

**Affiliations:** 1 Department of Pharmaceutics, School of Pharmacy, Qingdao University, Qingdao, 266021 People’s Republic of China; 2 National Engineering Research Center for Biomaterials, Sichuan University, Chengdu, 610064 People’s Republic of China

**Keywords:** Osteoarthritis, Reactive oxygen and nitrogen species, Thermo-sensitive hydrogels, Copper nanodots, Macrophage polarization

## Abstract

Osteoarthritis (OA) is a common degenerative joint disease that can lead to disability. Blocking the complex malignant feedback loop system dominated by oxidative stress and pro-inflammatory factors is the key to treating OA. Here, we develop a multifunctional composite thermo-sensitive hydrogel (HPP@Cu gel), which is utilized by Poloxamer 407 (P407) and hyaluronic acid (HA) mixture as the gel matrix, then physically mixed with copper nanodots (Cu NDs) and platelet-rich plasma (PRP). Cu NDs is a novel nano-scavenger of reactive oxygen and nitrogen species (RONS) with efficient free radical scavenging activity. HPP@Cu gel is injected into the articular cavity, where it form an in situ gel that slowly released Cu NDs, HA, and PRP, prolonging the duration of drug action. Our results indicate that HPP@Cu gel could efficiently remove RONS from inflammatory sites and promote repolarization of macrophages to an anti-inflammatory phenotype. The HPP@Cu gel therapy dramatically reduces cartilage degradation and inflammatory factor production in OA rats. This study provides a reliable reference for the application of injectable hydrogels in inflammatory diseases associated with oxidative stress.

**Graphical Abstract:**

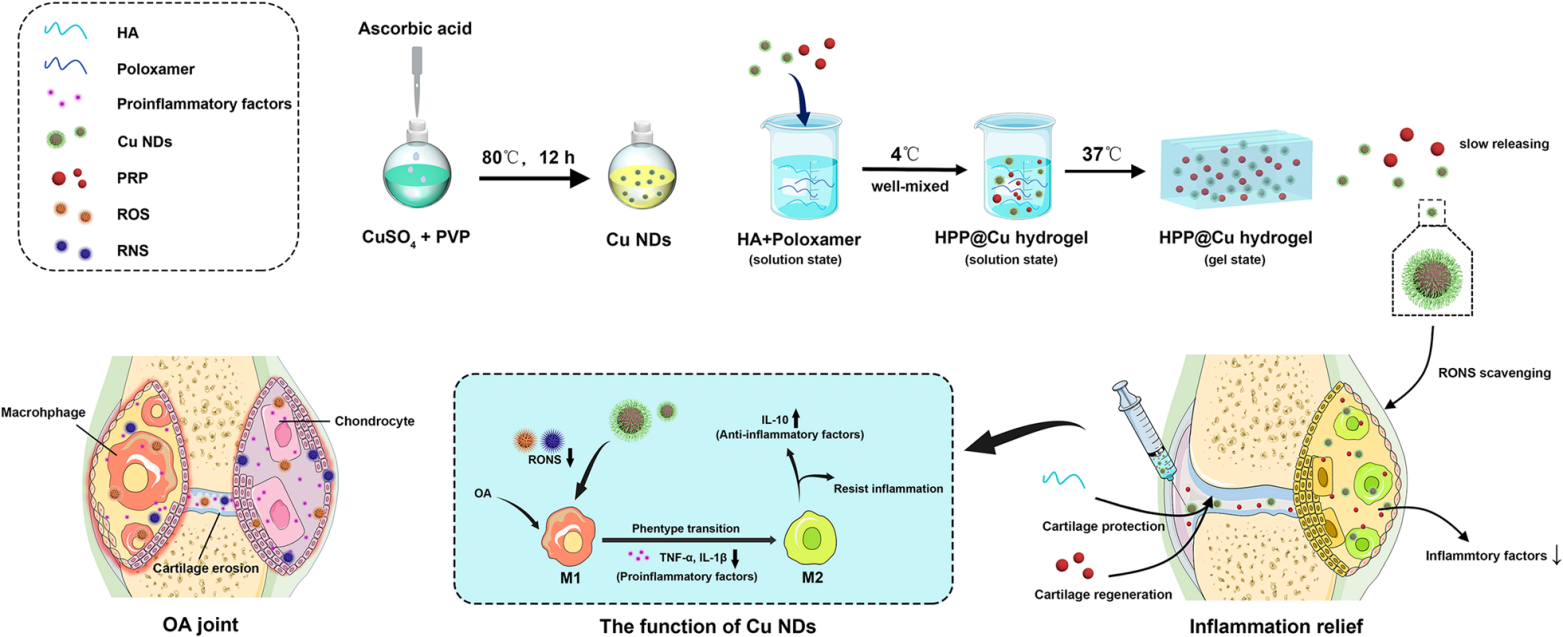

**Supplementary Information:**

The online version contains supplementary material available at 10.1186/s12951-022-01422-9.

## Introduction

Osteoarthritis (OA) is a common articular degenerative joint disease characterized by bone erosion, synovial inflammation, glycosaminoglycan loss, and cartilage destruction, which affects more than 15% of the global population [1–3]. Current early clinical treatment strategies of OA mainly focused on systemic or topical application of non-steroidal anti-inflammatory drugs (NSAIDs) to alleviate pain and inflammation. However, NSAIDs not only have short half-lives, fast clearance rates and require frequent dosing, but also lead to serious adverse effects from long-term administration, including peptic ulcer, renal failure and bleeding [4–6]. Although arthroplasty is considered to be an efficient treatment for advanced OA, the risks and expensive costs of the procedure must not be overlooked. Therefore, a therapeutic strategy that is safe and effective is highly desired to optimize the treatment of OA. Since the low systemic toxicity and long retention time at the joint, hydrogels of intra-articular (IA) injection have attracted a lot of attention [7–9]. Hydrogel has been demonstrated as a drug delivery system for maintaining long-term release, reducing the frequency of dosing [10–12]. Local injection administration reduces exposure and consumption to non-target sites, increases biocompatibility [13].

Studies have revealed that the inflammatory response of the OA joint cavity is largely dependent on excess reactive oxygen species (ROS) and reactive nitrogen species (RNS) in the articular cavity microenvironment, which can harm cells, proteins, lipids, and DNA in an irreversible way [14–18]. Exacerbation of reactive oxygen and nitrogen species (RONS) in the joint cavity provoked oxidative stress and worsens inflammation. To date, some small molecule drugs (such as vitamin C [19], *N*-acetylcysteine [20]) and nanoparticles [21] with antioxidant function had been developed and implemented for OA. Notably, many metals have been reported to have highly active antioxidant capacity due to their enzyme-like catalytic effects and caused widespread interest [22–27]. Copper (Cu), as one of the essential building blocks of many enzymes in the body such as Cu-Zn superoxide dismutase and tyrosinase, has a strong ability to trap free radicals in its ultra-small size nanocrystals [28, 29]. In addition, copper has a positive effect on promoting the production of cartilage matrix [30], facilitating osteogenesis and increasing bone strength [31].

In recent years, macrophages have been considered as a major target in many inflammatory disorders [32–34]. At the site of inflammation, the abnormal immune microenvironment promotes metabolic reprogramming of immune cells, prompting polarization of macrophages into the M1 phenotype. The mitochondrial respiratory activity of M1 macrophages is disrupted in the absence of an intact tricarboxylic acid cycle (TCA), resulting in a significant number of RONS that mediate the inflammatory response [35–38]. In addition, M1 macrophages could secrete NO and pro-inflammatory factors to induce and exacerbate inflammation [39–41]. In contrast, M2 macrophages with an intact TCA system and normal mitochondrial respiratory function could mediate immunosuppression and tissue repair via secreting anti-inflammatory factors and chondrogenic factors [42, 43]. In this regard, Lin et al. fabricated a copper-incorporated BGC (Cu-BGC) scaffold through triggered cartilage immune response to anti-arthritis [44]. Cu-BGC scaffolds were authenticated to induce macrophage polarization toward M2 type and down-regulate the expression of inflammatory factors. Inspired, we synthesized copper nanodots (Cu NDs) as a microenvironmental purifier of biological tissues, which has efficient RONS scavenging properties and promotes reprogramming of M1 macrophages toward the M2 phenotype, exerting anti-inflammatory effects.

**Fig. 1:**
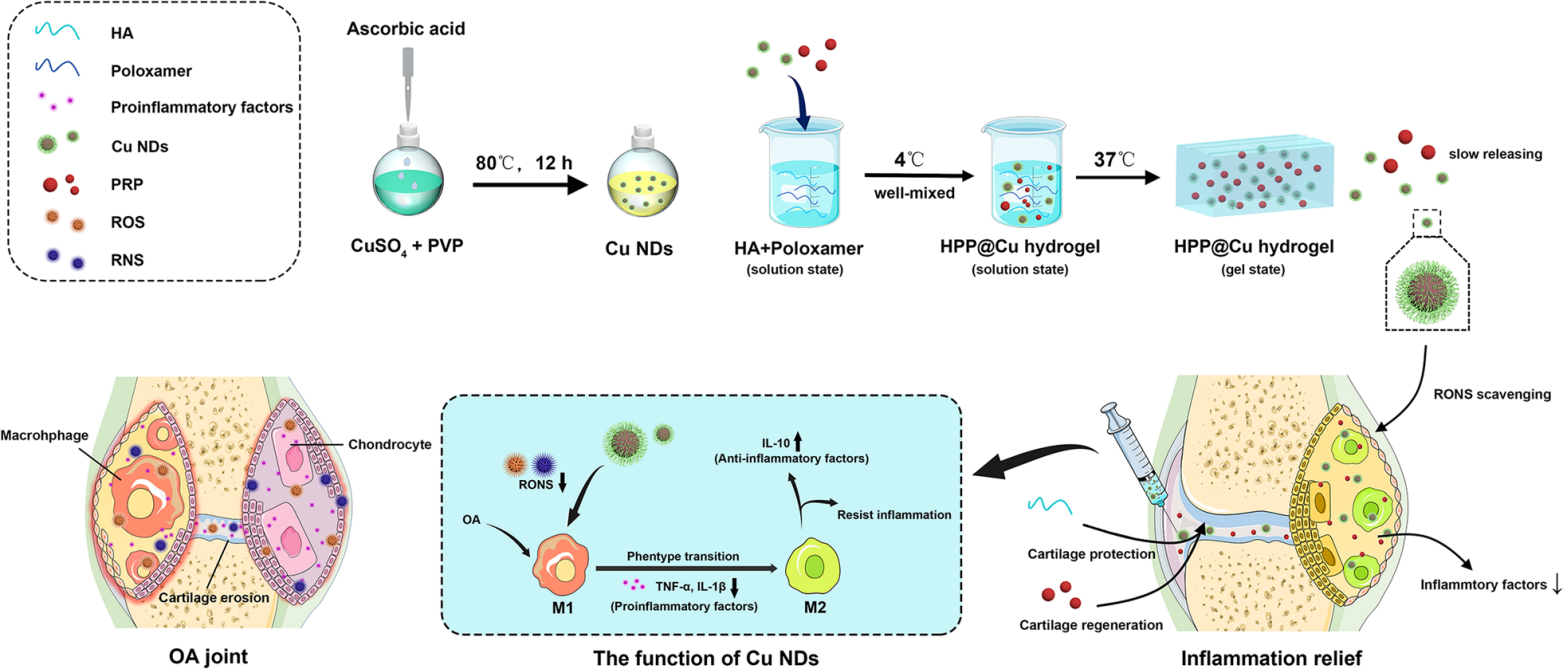
Schematic illustration for the synthesis processes of HPP@Cu thermoresponsive hydrogel and treatment mechanism as articular microenvironment purifier for OA

Based on the consideration of the short retention period of nanodots in the joint, we finally developed a multifunctional composite thermo-sensitive hydrogel system for intra-articular injection to treat OA (Fig. 1). A mixture of hyaluronic acid (HA) and poloxamer 407 (P407) was employed as a thermo-sensitive gel matrix. HPP@Cu hydrogel was prepared by physically adding Cu NDs and platelet-rich plasma (PRP) to the gel matrix. HA is a natural, non-immunogenic glycosaminoglycan present in the extracellular matrix and plays an essential role in maintaining the phenotype of chondrocyte and viscoelasticity of synovial fluid [45]. PRP is a natural reservoir of many cytokines and growth factors, and has a proliferative effect on chondrocytes [46]. HPP@Cu hydrogel was injected into the joint in the form of a solution, which underwent phase transformation into hydrogel at body temperature and slowly released Cu NDs, HA and PRP. As shown in Fig. 1, HPP@Cu hydrogel combines the best of each component, clearing RONS and promoting the polarization macrophages to M2-type, and inhibit the production of inflammatory factors. Moreover, HPP@Cu hydrogel protects cartilage from damage and promotes cartilage regeneration, effectively blocking the vicious cycle and self-perpetuation of OA. The study may be a reliable reference for the rational design of anti-inflammatory materials and opens up new perspectives for the treatment of OA.

## Results and discussion

### Physical characterizations of Cu NDs

**Fig. 2:**
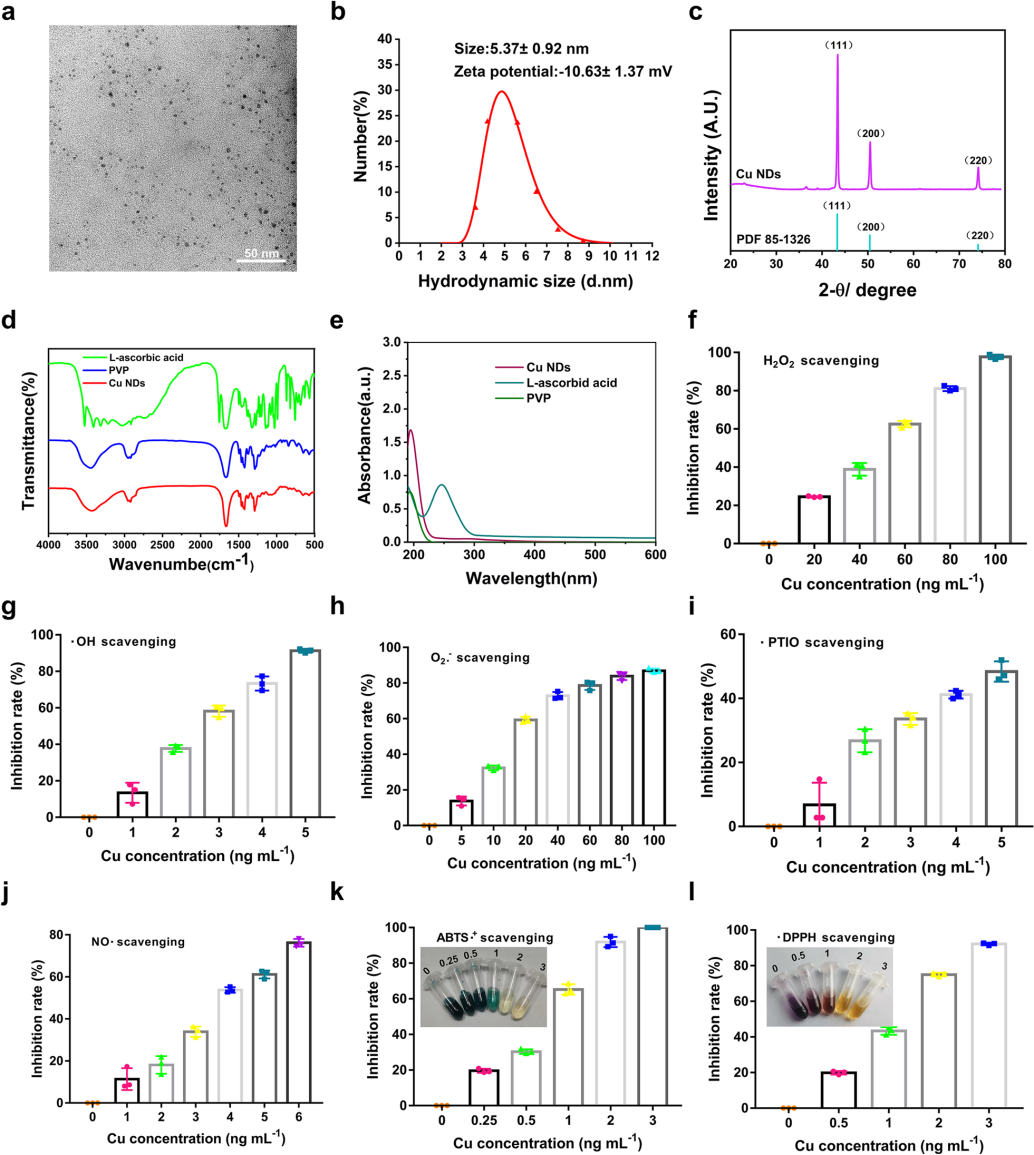
Physical characterizations and RONS scavenging activity of Cu NDs. **a** TEM image of Cu NDs. Scale bars = 50 μm. **b** Hydrodynamic diameter distribution of Cu NDs. **c** XRD pattern of Cu NDs. **d** FTIR spectra of Cu NDs. **e** UV-*vis* spectra of Cu NDs. **f** H_2_O_2_ scavenging activity of Cu NDs at various concentrations. **g** NO· scavenging activity of Cu NDs at various concentrations. **h** ·O_2_^-^ scavenging activity of Cu NDs at various concentrations. **i** ·PTIO scavenging activity of Cu NDs at various concentrations. **j** NO· scavenging activity of Cu NDs at various concentrations. **k** ABTS·^+^ scavenging activity of Cu NDs at various concentrations. The inset is ABTS·^+^ test solution treated with various concentrations of Cu NDs. **l** ·DPPH scavenging activity of Cu NDs at various concentrations. The inset is ·DPPH solution treated with various concentrations of Cu NDs. In **f–l**, data represent means ± SD from three independent replicates

The Cu NDs with ultra-small particle size was prepared by hydrothermal reduction method [28, 47]. Morphological characteristic of Cu NDs was observed by transmission electron microscopy (TEM). As shown in Fig. 2a, the particle size of Cu NDs was small, with an average diameter of ~ 5 nm. The particle size was homogeneous and there was no obvious aggregation of nanoparticles. Dynamic light scattering (DLS) revealed that the hydrodynamic diameter of Cu NDs was 5.37 ± 0.92 nm, and the particle size distribution curve was in accordance with Gaussian normal distribution (Fig. 2b). Cu NDs exposed more active sites owing to its smaller dimension and higher specific surface area, which could guarantee excellent antioxidant activity [28]. Zeta potential analysis showed that *ζ*-potential of Cu NDs was − 10.63 ± 1.37 mV. Stabilization experiments in vitro (Additional file 1: Fig. S1) showed that, Cu NDs was stable in fetal bovine serum (FBS) and medium supplemented with 10% FBS for at least 48 h and stable in water and PBS for at least 4 weeks. The oxidation state of Cu was studied by powder X-ray diffraction. The X-ray diffraction (XRD) pattern (Fig. 2c) revealed that all the peaks of the synthesized nanoparticles matched with the standard card of Cu (PDF 85-1326). Three intense diffraction peak signals at 2*θ* = 43.3°, 50.4°, and 74.12°, correspond to the (111), (200), and (220) crystallographic planes of face-centered cubic copper, respectively. These results showed that the crystalline composition of Cu NDs was only copper monomer and there was no presence of copper in the oxidation state. Moreover, polyvinyl pyrrolidone (PVP) was used as a capping agent to inhibit crystal growth during the synthesis of Cu NDs. PVP was proven to be non-toxic, non-sensitizing and non-irritating, and therefore it is widely used in food and pharmaceutical applications. The structural analysis of Cu NDs by Fourier transform infrared (FTIR, Fig. 2d) revealed that PVP was successfully coated on the surface of the nanoparticles, which could be further verified by ultraviolet-visible (UV-*vis*) spectrum (Fig. 2e). The above results demonstrated that we successfully prepared Cu NDs with good stability.

### RONS scavenging assays of Cu NDs

Under ultra-small size conditions, metal nanoparticles exhibit attractive properties such as variable fluorescence with size and discrete electronic states due to their strong quantum binding effect on electrons. Previously, Cu nanoparticles or nanoclusters with ultra-small dimensions had been shown to have the ability to catalyze H_2_O_2_ and superoxide anion (·O_2_^-^) with peroxidase-mimicking activity [28]. Herein we investigate the RONS scavenging activity of Cu NDs by colorimetric assay. As shown in Fig. 2f–l, Cu NDs showed high RONS scavenging activity in a concentration-dependent manner. Approximately 97% of H_2_O_2_ and 87% of ·O_2_^-^ were removed by 100 ng/mL of Cu NDs, and 91% of ·OH and 61% of NO· were scavenged by 5 ng/mL of Cu NDs. Also, ABTS radicals, PTIO radicals, and DPPH radicals were used to evaluate the free radical scavenging ability of Cu NDs. The results showed that almost all ABTS radicals and 92% of DPPH radicals were easily scavenged by Cu NDs at a concentration of 3 ng/mL. A total of 48% of PTIO radicals could be scavenged in the presence of Cu NDs at a concentration of 5 ng/mL. The above results suggested that Cu NDs exhibited extensive RONS scavenging ability at very low concentrations, which may be due to its smaller particle diameter exposing a larger fraction of active sites.

In addition, to monitor the scavenging process of free radicals, the UV Absorption Spectrum of ABTS radicals and DPPH radicals during co-incubation with Cu NDs were scanned separately. The results suggested that Cu NDs scavenged free radicals in a time-dependent manner and that Cu NDs was able to perform most of the removal tasks in 60 min (Additional file 1: Fig. S2).

### Preparation and rheological study of HPP@Cu gel

**Fig. 3:**
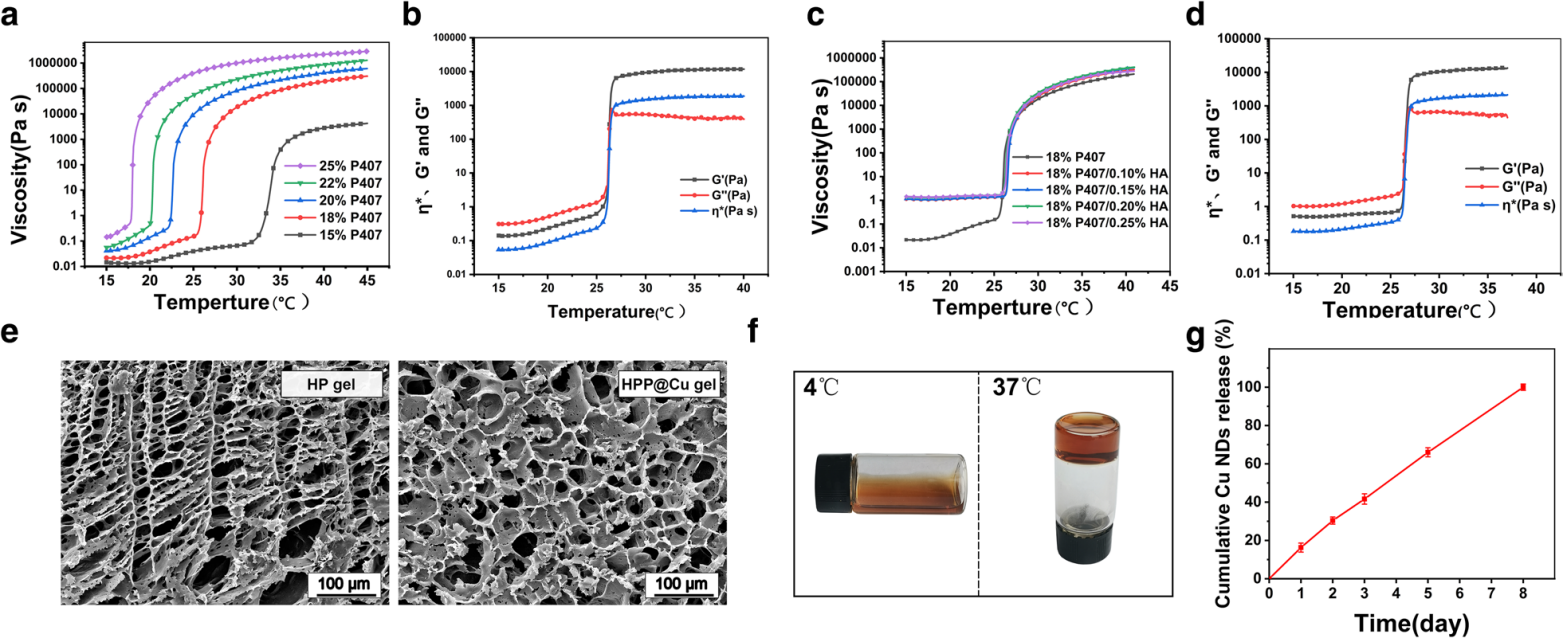
**a** The viscosity profile of the P407 hydrogels at various concentrations (15%, 18%, 20%, 22%, 25%) altered with increasing temperature. **b** Variation of viscoelastic parameters (η*, *G’*, *G’’*) of 18% P407 at different temperatures. **c** The viscosity profile of the P407 hydrogels (18%) after adding HA at various concentrations (0.1%, 0.15%, 0.2%, 0.25%) altered with increasing temperature. **d** Variation of viscoelastic parameters (η*, *G’*, *G’’*) of 18% P407 added with 0.20% HA at different temperatures. **e** SEM of HP gel and HPP@Cu gel. Scale bars = 100 μm. **f** Images of HPP@Cu gel at 4 ℃ or 37 ℃. **g** Release profile of Cu NDs loaded by HPP@Cu gel. Data represent means ± SD from three independent replicates

The HPP@Cu composite thermo-sensitive hydrogels were prepared by physical blend method. First, the viscosity curves and viscoelastic curves of various concentrations of P407 with temperature were measured by rheometer, and the gelation temperature was recorded. As the results in Fig. 3a showed, the *η* of the gel varied with temperature, which revealed the temperature sensitivity of the material. *η* increased with the concentration of P407, so 15% of P407 had the smallest *η*. In rheological testing, *G’* represents the ability of a material to spring back after deformation and is often used to characterize the solid-like behavior of a material. While *G’’* is the energy dissipated by heat dissipation when the material deforms, and is usually used to characterize the liquid-like behavior of a material. When *G’* is equal to *G’’*, the gel undergoes a phase change, and the temperature at this point is the gelation temperature. The gelation temperature of 15% of P407 was around 37 °C, which did not guarantee that it would gel at body temperature (Additional file 1: Fig. S3a-b). Therefore, under the premise of ensuring that the gel can be gelated in vivo, 18% P407 was finally selected was selected for the following gel preparation, because its lower viscosity can accommodate the introduction of more HA. The results (Fig. 3c and Additional file 1: Fig. S3c, d) were shown that the addition of HA did not influence the gelation temperature and gelation time. Undesirably, as the HA concentration increased, the *η* of the composite gel also increased at low temperatures. In addition, the composite hydrogel should have a suitable viscosity for injection, as well as sufficient strength to retain its shape to retard the in situ release of the drug. Ultimately, 0.2% HA/18% P407 were selected as the final thermo-sensitive hydrogel matrix, which had a gelation temperature of about 25 °C and a gelation time of about 40 s (Fig. 3d and Additional file 1: Fig. S3c, d).

### Morphological characterization of HPP@Cu gel

HPP@Cu gels were prepared by adding the contents by a simple physical blending method. HPP@Cu gels existed as a solution at 4 °C and changed from the solution state phase to the gel state as the temperature increased, and this transition was due to the thermo-sensitive nature of the P407 (Fig. 3f). To examine the internal morphology of the gels, the HPP@Cu thermo-sensitive gels were lyophilized at low temperatures and then observed by SEM. As illustrated in Fig. 3e, both the composite thermo-sensitive hydrogels before and after the addition of Cu NDs and PRP showed irregular porous structures, which were favorable for the growth and adhesion of chondrocytes.

### Study on the dissolution behavior of HPP@Cu gel and the release behavior of Cu NDs, HA, and PRP-derived TGF-β1 from HPP@Cu gel

The uniform and slow dissolution of HPP@Cu gel is the basis for the slow release of Cu NDs, HA, and PRP. Then, the time that the HPP@Cu gel could maintain the long-term drug release process was investigated by the dissolution experiment. As show in Additional file 1: Fig. S4, the HPP@Cu gel was slowly dissolved in the release medium until it was completely dissolved on day 8.

The release behavior of Cu NDs, HA, and RPR from HPP@Cu gel was investigated by release experiments in vitro. Cu NDs were slowly and continuously released from the gel (Fig. 3g). At day 3, Cu NDs were released from the gel by 40%. The concentration of HA in the release medium was determined by CTAB turbidimetric method [48]. The CTAB turbidimetric method is a specific method capable of detecting the content of hyaluronic acid in a cross-linked state in a complex environment. As shown in Additional file 1: Fig. S5, HA was released slowly in the HPP@Cu gel and no abrupt release behavior was observed. The cumulative release of HA from the gel reached 41% on day 3. On day 8 the gel was completely dissolved resulting in complete release of HA. RRP is a mixture containing several cytokines and growth factors, including TGF-β1. The release behavior of PRP from the HPP@Cu gel was examined indirectly by assaying the concentration of PRP-derived TGF-β1 in the release medium. As shown in Additional file 1: Fig. S6, TGF-β1 was slowly released in the gel until day 8.

Despite the different properties of Cu NPs, HA, and PRP (e.g., molecular weight size, dimensional size, and hydrophilic properties), they showed similar release behaviors from the HPP@Cu gel. Combining the results of dissolution experiments and the content release experiments of gel in vitro, it is seen that the cumulative release behavior of Cu NDs, PRP-derived TGF-β1, and HA from the HPP@Cu gel is similar to the dissolution behavior of the HPP@Cu gel. The phenomenon suggested that the dissolution of the HPP@Cu gel controlled the release of the contents. That might be due to interactions such as hydrogen bonding, cross-linking, and electrostatic attraction formed between Cu NDs, HA, PRP-derived protein molecules and P407, which allows them to be released from the gel in a slow desorption manner [49–52].

### RONS scavenging assays of HPP@Cu gel

Simultaneously, the free radical scavenging capacity of the HPP@Cu composite thermo-sensitive hydrogel was examined by the UV chromogenic method. The results showed that HPP@Cu gel had a similar free radical scavenging ability as Cu NDs (Additional file 1: Fig. S7).

### Cytotoxicity evaluation of HPP@Cu gel in vitro

**Fig. 4:**
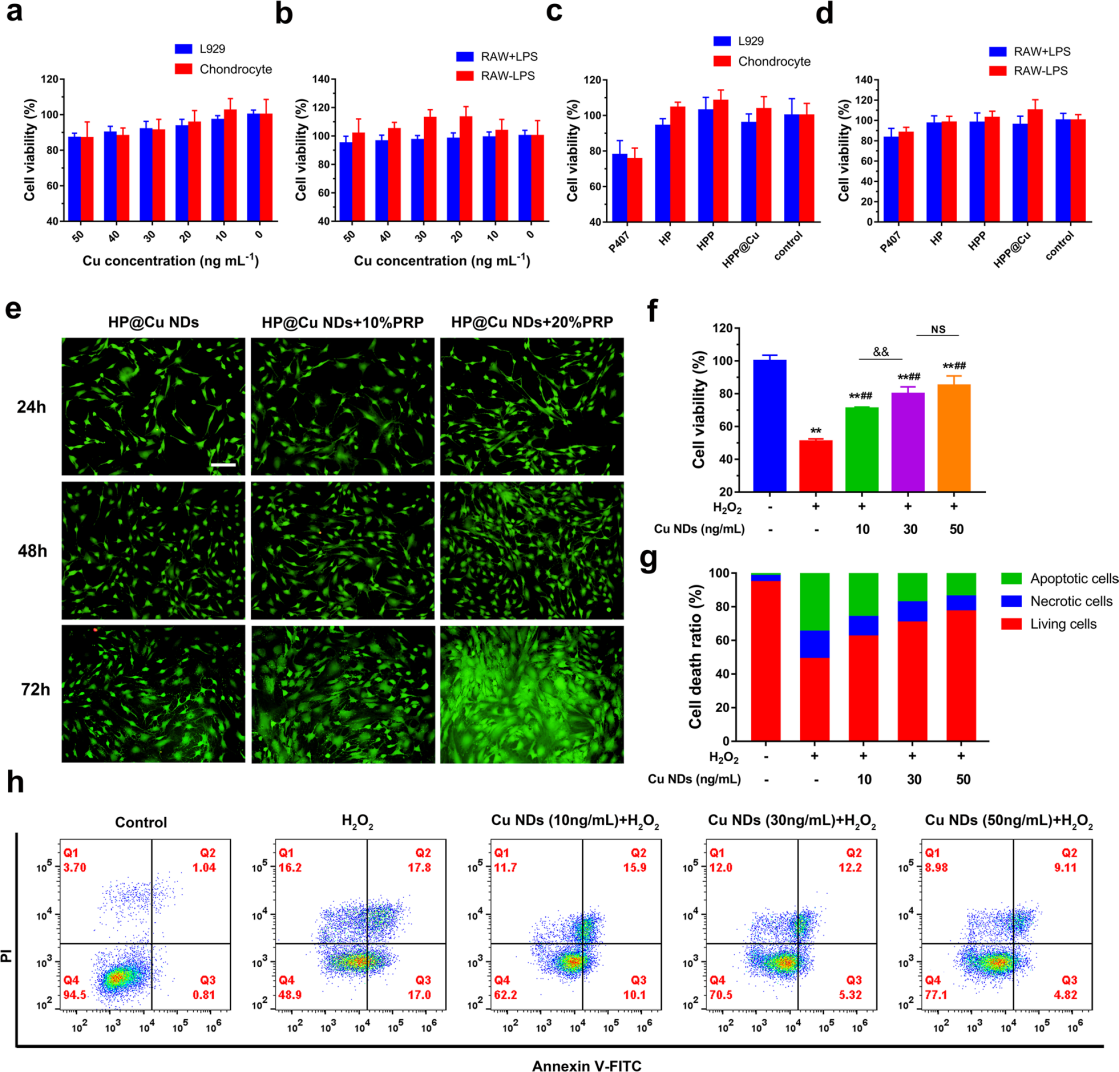
**a** Cytotoxicity of Cu NDs on L929 and chondrocyte. **b** Cytotoxicity of Cu NDs on activated RAW264.7 and inactivated RAW264.7. **c** Cell viability of L929 and chondrocyte with different treatments. **d** Cell viability of activated RAW264.7 and inactivated RAW264.7 with different treatments. **e** Fluorescence images of Calcein-AM/PI staining of chondrocyte after incubation with different concentrations of PRP in HPP@Cu gel. Scale bars = 100 μm. **f** Cell viabilities of chondrocyte under different treatment conditions. Data represent means ± SD, n = 5. **p < 0.01, ##*p* < 0.01, and &&*p* < 0.01. NS means not significant. **g** Quantification of the ratio of apoptotic and living cells by flow cytometry analysis according (**h**). **h** Flow cytometry analysis of apoptotic and necrotic cells under different treatment conditions. In **a–d**, data represent means ± SD, n = 5

Using L929, RAW264.7, and rat chondrocytes as model cells, we investigated the cytotoxicity of HPP@Cu gel and Cu NDs on normal cells, immune cells, and chondrocytes. According to the results of MTT experiments, the cell survival rate of Cu NDs was above 80% (Fig. 4a, b), which indicated the good biocompatibility of Cu NDs at low concentration (0–50 ng/mL). Such results are consistent with previous studies that copper nanoparticles are usually non-toxic at concentrations less than 5 µg/mL [28, 53]. The results in Fig. 4c,  d showed that there was no significant difference between the HPP@Cu gel and the control group, which illustrated the safety of the composite thermo-sensitive gel material. Notably, cell survival in the HP and HPP groups was elevated compared to the P407 group, implying good biocompatibility of PRP and HA, which was in accordance with the results of previous literature [45, 54, 55].

### Cell proliferation of HPP@Cu gel

PRP is rich in several growth factors and cytokines such as platelet-derived growth factor (PDGF), transforming growth factor-β (TGF-β), insulin-like growth factor (IGF), which are known to promote chondrocytes proliferation [56]. Calcein-AM/PI was used to assess the proliferative effect of chondrocytes. As shown in Fig. 4e, the cell density in the composite hydrogel group with the addition of PRP tended to increase compared to the composite hydrogel group without the addition of PRP. Among them, the composite thermo-sensitive hydrogel with 20% of PRP added had the strongest pro-chondrocyte proliferation ability. In addition, the few dead cells in the field of view further demonstrated the cytocompatibility of the HPP@Cu composite thermo-sensitive hydrogel.

### RONS scavenging activity assessment in vitro

**Fig. 5:**
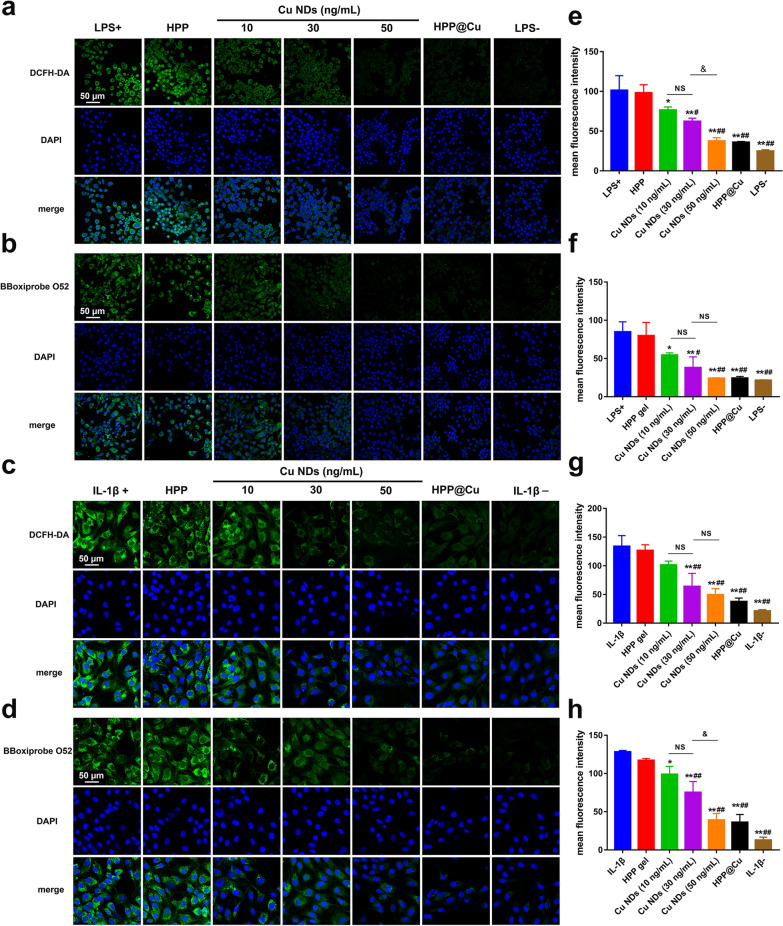
RONS scavenging capacity of HPP@Cu gel for RAW264.7 and chondrocyte. **a** Confocal fluorescence images of the levels of ROS of RAW264.7. **b** Confocal fluorescence images of the levels of RNS of RAW264.7. **c** Confocal fluorescence images of the levels of ROS of chondrocyte. **d** Confocal fluorescence images of the levels of RNS of chondrocyte. **e–h** Corresponding to **a–d**, the mean fluorescence intensity was quantified by ImageJ, respectively. In **e–h**, data represent means ± SD, n = 3. *p < 0.05, #*p* < 0.05, and &*p* < 0.05. **p < 0.01, ##*p* < 0.01, and &&*p* < 0.01. NS means not significant

In the OA pathological environment, activated macrophages with altered energy metabolism produces a large number of RONS, which could damage chondrocytes and further amplify the inflammatory response [16, 21, 57]. Therefore, inhibition of RONS levels in macrophages and chondrocytes plays a crucial role in delaying the progression of OA. 2′-7′-dichlorodihydrofluorescein diacetate (DCFH-DA) was employed as a ROS probe to monitor the total ROS scavenging activity of Cu NDs in vitro, then we employed BBoxiProbe® O13 as a RNS probe to detect the total RNS scavenging activity of Cu NDs in vitro. In the present study, lipopolysaccharide (LPS)-activated macrophages and exogenous IL-1β-stimulated chondrocytes were used as in-vitro inflammatory cell models. As presented in Fig. 5a–d, RONS levels were markedly elevated in LPS-treated RAW264.7 and IL-1β-treated chondrocytes, whereas RONS levels were significantly reduced in cells pretreated with Cu NDs or HPP@Cu gels. Moreover, fluorescence intensity quantitative analysis of RONS by Image J showed that Cu NDs scavenged RONS in a concentration-dependent manner (Fig. 5e–h). The efficient RONS scavenging activity of Cu NDs in vitro was further demonstrated by flow cytometry analysis (Additional file 1: Fig. S8).

### Protective effects of Cu NDs on H_2_O_2_-induced chondrocytes damage

Chondrocytes, the key cells in cartilage, act as accelerators of cartilage degradation and pro-inflammatory agents when damaged [58]. Considering the fact that large amounts of RONS impair mitochondrial function and lead to chondrocytes death, the notion that protecting chondrocytes from damage by RONS could reduce chondrocytes mortality is plausible. Here we exposed chondrocytes pretreated with Cu NDs to H_2_O_2_ to investigate the protective effect of Cu NDs. The results of the MTT assay (Fig. 4f) showed that H_2_O_2_ caused chondrocytes apoptosis and reduced the low survival rate to 51%. Whereas the survival rate of chondrocytes pretreated with Cu NDs (50 ng/mL) elevated to 85% because of consumption of RONS. Moreover, Cu NDs in the concentration range of 10–50 ng/mL protected chondrocytes from H_2_O_2_ in a concentration-dependent manner. Finally, the percent of apoptotic cells and viable cells caused by H_2_O_2_ was measured by flow cytometry. As Fig. 4g, h showed that, the chondrocyte survival rate was only 48.9% and the apoptosis rate reached 34.8% after H_2_O_2_ stimulation. In comparison, the percentage of viable cells increased dramatically after Cu NDs treatment, which further confirmed the protective effect of Cu NDs on chondrocytes.

### Phenotype transition study of macrophages in vitro

**Fig. 6:**
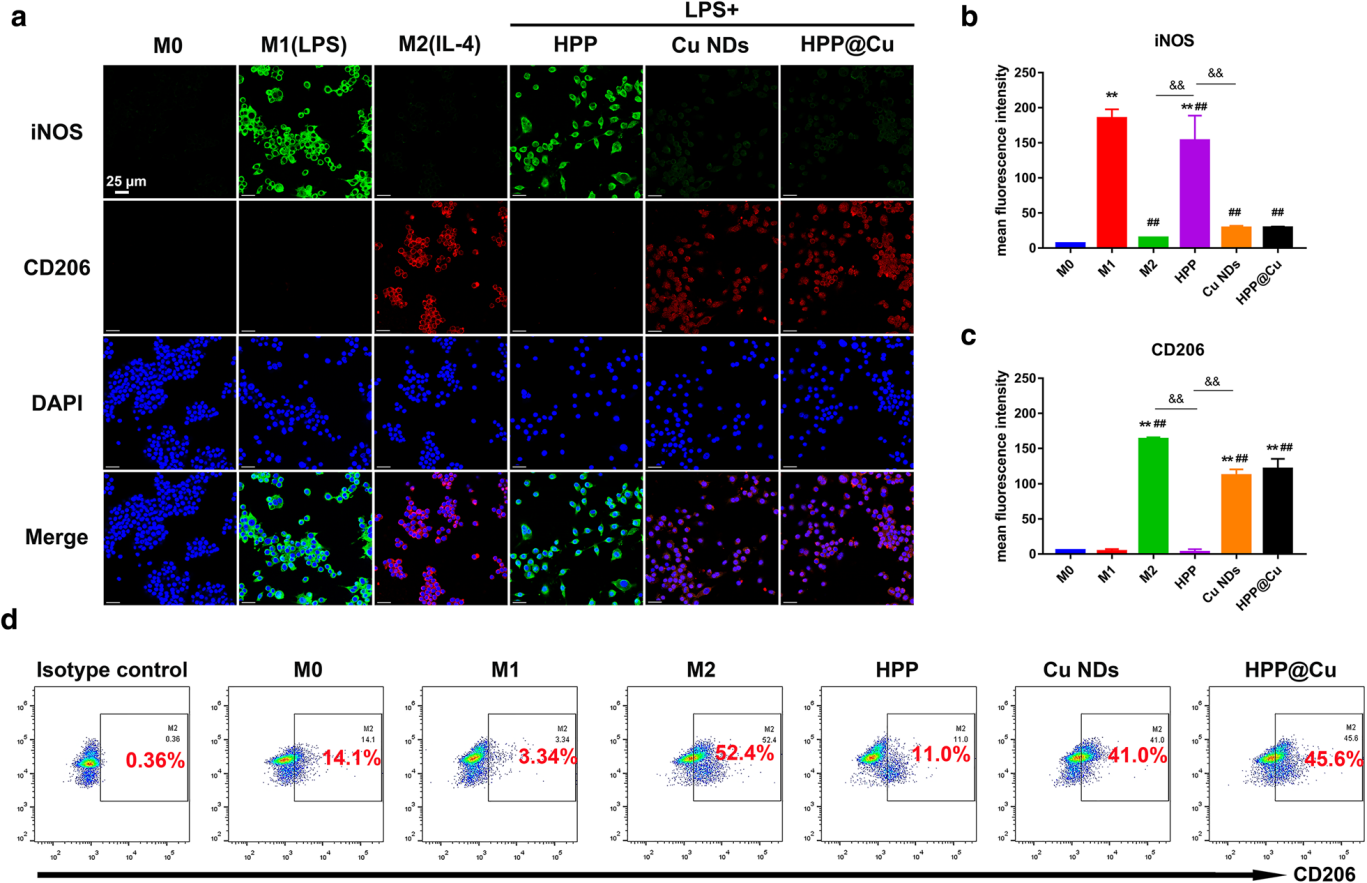
Effect of HPP@Cu gel on macrophage phenotype. **a** Immunofluorescence analysis of markers of M1 (iNOS, green) and M2 (CD206, red) on macrophages after different treatments. **b** Corresponding to **a**, the mean fluorescence intensity of iNOS was quantified by ImageJ. **c** Corresponding to **a**, the mean fluorescence intensity of CD206 was quantified by ImageJ. **d** Flow cytometry analysis of the percentage of M2 in the macrophage population. In **b**, **c**, data represent means ± SD, n = 3. **p < 0.01, ##*p* < 0.01, and &&*p* < 0.01. NS means not significant

Extensive infiltration of macrophages in synovial tissue is a feature of OA [59]. Macrophages are a class of intrinsic immune cells with plasticity, whose phenotype and function are regulated by the surrounding environment [60]. Macrophages are mainly divided into two phenotypes, the pro-inflammatory M1 type and the anti-inflammatory M2 type. M1 macrophages can be differentiated by LPS stimulation and secrete various pro-inflammatory factors, such as IL-1β, IL-6, and TNF-α, triggering an inflammatory cascade response. M2 macrophages can be differentiated by interleukin-4 (IL-4) stimulation and secrete various anti-inflammatory factors such as IL-10 and TGF-β, exerting anti-inflammatory and immunomodulatory effects. It has been found that reprogramming the phenotype of macrophages is an effective strategy for the treatment of OA [61]. The ability of Cu NDs to phenotypically reprogram macrophages was studied by immunofluorescence staining of RAW264.7 for iNOS (M1 marker) and CD206 (M2 marker). Confocal results (Fig. 6a–c) showed that untreated RAW264.7 did not exhibit bright fluorescence in the cytoplasm due to low expression of iNOS and CD206 receptors. LPS could induce RAW264.7 polarization into M1-type macrophages with high expression of iNOS receptors, and IL-4 could induce and M2-type macrophages with high expression of CD206 receptors. As we expected, the expression of CD206 was significantly increased after treating activated macrophages with Cu NDs for 24 h, which implied that Cu NDs could program macrophages from M1 to M2. Subsequently, this trend was observed by flow cytometric analysis to detect the percentage of M2 in the total macrophage population. As shown in Fig. 6d, Cu NDs could cause a significant increase in the percentage of M2 macrophages, which further validated the phenotypic reprogramming effect of Cu NDs on macrophages.

### The degradation experiment in vivo

For the purpose of studying the degradation time of HPP@Cu hydrogel in vivo, Bright blue colored HPP@Cu hydrogel was injected into the joint cavity of rats. The joints were dissected at 1 h, 3 day and 5 day after injection to observe the degradation of the gel. Brilliant blue dye is a synthetic pigment that is widely used in the food, medicine and cosmetic industries [62, 63]. As shown in Additional file 1: Fig. S9, the addition of Bright blue allowed for a clear macroscopic view of the gel. At 1 h after injection, the HPP@Cu gel was filled throughout the joint cavity. The gel was observed in the femur, tibia, and joint capsule. On day 3 after the injection, the gel degraded in the joint cavity causing the gel content to decrease. By day 5, the gel was difficult to find in the joint cavity.

At each time point, the entire joint was homogenized and assayed for Cu NDs. As shown in Additional file 1: Fig. S10,  the amount of Cu NDs remaining in the joint on day 3 was 56%. By day 5, the amount of Cu NDs in the joint was only 2%, which indicated that the gel was almost completely degraded and metabolized in the joint cavity. Collectively, these data above suggested the HPP@Cu gel was almost degraded within 5 days in the joint.

### Therapeutic effect on MIA-induced rat OA models

**Fig. 7:**
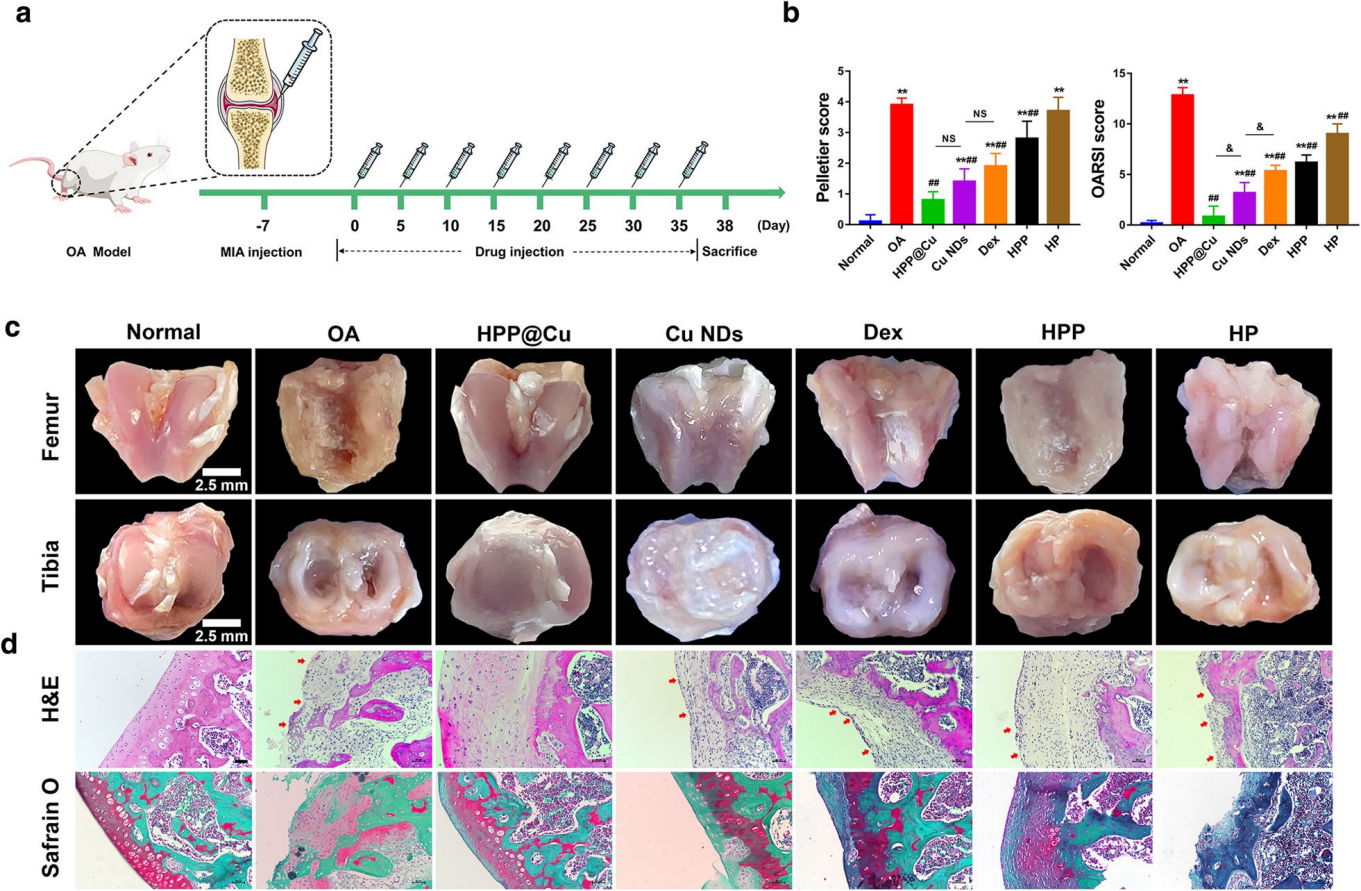
Histological analysis after treatment in vivo. **a** Schematic illustration of the establishment and treatment schedule of MIA-induced OA rats. **b** Pelletier score analysis according macroscopic appearance, and OARSI score analysis according H&E staining and Safranin O staining. Data represent means ± SD, n = 3. &*p* < 0.05. **p < 0.01, and ##*p* < 0.01. NS means not significant. **c** Macroscopic appearance of joints after treatment. **d** H&E staining and Safranin O staining of joints after treatment. Scale bars = 50 μm

We assessed the therapeutic efficacy of the HPP@Cu gel on Sodium iodoacetate (MIA) induced rats according to the therapeutic scheme outline shown in Fig. 7a. To assess the curative effect of HPP@Cu gel on OA, MIA was injected intra-articularly into the right hind limb of rats to induce an OA model. After 7 days of induction of the OA model, the rats were grouped and treated. All rats were administered every 5 days until sacrifice on day 38. The joint tissues of the right hind limb of rats were dissected, observed visually, and the morphology of the joint was assessed macroscopically according to the Pelletier scoring system [64]. As shown in Fig. 7c, the femur and tibia of the articular surfaces in control group did not have any damage, and the cartilage surfaces were smooth and flat. In contrast, the OA model group showed severe bone erosion and fissures in both femoral plateau and tibial plateau, and the cartilage surface was severely damaged. As we expected, the articular cartilage of the rats in the HPP@Cu group was similar to that of the normal group, with almost no damage. Dexamethasone is one of the first-line anti-inflammatory drugs that is widely used in the clinical treatment of OA [65, 66]. Intra-articular injection of dexamethasone can relieve joint pain and delay inflammation. Based on the known therapeutic effects of dexamethasone, dexamethasone injection was therefore used as a positive control drug in this study to examine the efficacy of HPP@Cu gel formulation. Due to the rapid clearance of the joint cavity, there was slight damage of articular cartilage in the Cu NDs group and Dexamethasone (Dex) group, with rough cartilage surfaces and minor localized erosions. The HPP and HP groups had more severely damaged articular cartilage, proliferation of osteophyte, and varying degrees of bone erosion.

The Pelletier scoring system is commonly used to score the degree of degeneration of the articular surfaces of macroscopic specimens [64, 67]. The higher the score, the more severe the cartilage damage on the joint surface. The Pelletier scale has 5 levels. A score of 0 is given for an intact articular surface with normal color. Score 1 is given for a rough, slightly gray articular surface with small fissures. A score of 2 is given for an eroded articular surface with cartilage defects deep to the superficial or middle cartilage layer. An ulcerated articular surface with deep cartilage defects is scored as 3. A score of 4 is given for a joint of which the cartilage surface is stripped and the subchondral bone is exposed. The results of the Pelletier score (Fig. 7b) showed that the Pelletier score was significantly higher in the OA group compared with the control group. Compared with the OA group, all administration groups were able to significantly reduce the Pelletier score and improve cartilage damage. Among all the administration groups, the HPP@Cu group had the lowest Pelletier score, which indicated that HPP@Cu gel had a good effect on the repair of the joint surface.

Moreover, the therapeutic effect of OA was evaluated by hematoxylin and eosin (H&E) and Safranin-O fast green staining, and the staining results were evaluated based on Osteoarthritis Research Society International (OARSI) scoring standard [68, 69]. H&E staining result (Fig. 7d) showed that the cartilage matrix damage reached the calcified layer and the chondrocyte arrangement was disturbed in the OA group. Results of the Safranin-O fast green staining showed that one of the typical features of OA is the loss of proteoglycans. Moreover, the joints in the HPP and HP groups exhibited similar pathobiological conditions to those in the OA group. In contrast, the joints treated with HPP@Cu gel showed significantly less damage, as evidenced by the orderly arrangement of chondrocytes, the absence of fissures on the cartilage surface and the uniform distribution of proteoglycans. In addition, the OARSI score (Fig. 7b) further illustrated the potential of HPP@Cu gel to block the degradation of articular cartilage and glycosaminoglycans. Given that the slow release of the gel system contributed to a longer drug duration of action, the HPP@Cu thermo-sensitive hydrogel platform scored lower than the Cu NDs and Dex groups.

**Fig. 8:**
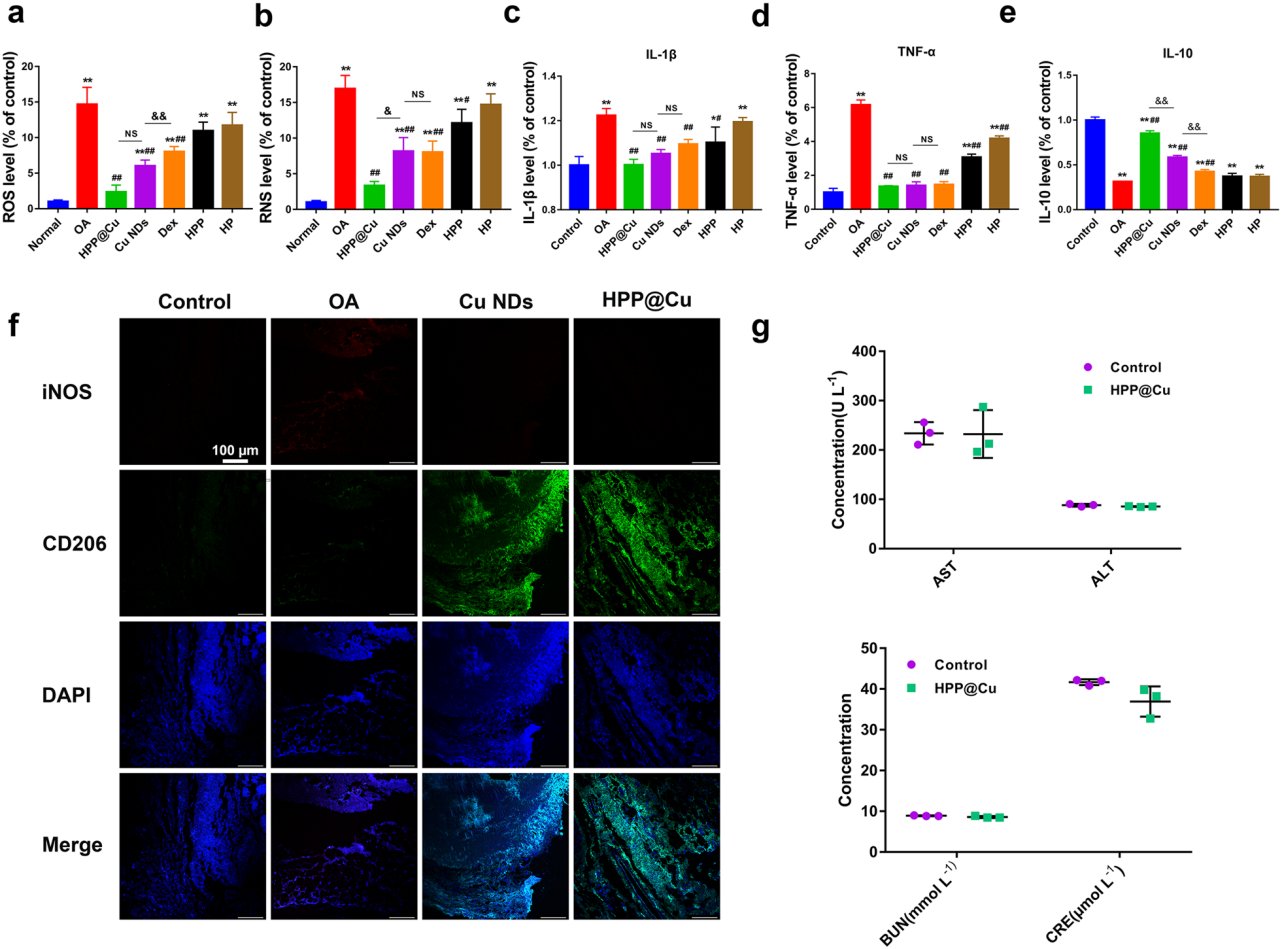
**a** Levels of ROS in joint tissue homogenate. **b** Levels of RNS in joint tissue homogenate. **c** Levels of IL-1β in joint tissue homogenates. **d** Levels of TNF-α in joint tissue homogenates. **e** Levels of IL-10 in joint tissue homogenates. **f** Immunofluorescence staining of iNOS (M1 marker, red) and CD206 (M2 marker, green) of synovial tissue. **g** Liver function indicators levels and kidney function indicators levels of serum after treatment. In **a–e**, and **g**, data represent means ± SD, n = 3. *p < 0.05, #*p* < 0.05. **p < 0.01, ##*p* < 0.01, and &&*p* < 0.01. NS means not significant

In addition, we measured the ROS and RNS levels in cartilage tissue homogenates using BBoxProbe® O13 and BBoxProbe® O52, respectively. As shown in Fig. 8a, b, the levels of ROS and RNS were significantly increased in the OA group compared with the control group. In contrast, ROS and RNS levels in cartilage treated with HPP@Cu gel were significantly decreased, which suggested that HPP@Cu gel could effectively remove RONS from the joint. The phenotypic effects of HPP@Cu gel on synovial macrophages were subsequently assessed by subjecting synovial tissue to immunofluorescence staining for iNOS (M1 marker) and CD206 (M2 marker). Compared with the control group (Fig. 8f), iNOS levels were significantly higher in the joints of the OA group, while iNOS levels were reduced to varying degrees after HPP@Cu gel treatment or Cu NDs treatment, which suggested that HPP@Cu gel and Cu NDs could efficiently promote the phenotypic transition of macrophages from M1 to M2.

It was reported that M1 macrophages of the OA microenvironment could secrete large amounts of pro-inflammatory factors through immune activation, which could again induce inflammation and trigger an inflammatory cascade response [70–72]. Finally, we further assessed the treatment effect by measuring pro-inflammatory factors (TNF-α, IL-1β) and anti-inflammatory factors (IL-10) in cartilage tissue homogenates. As shown in Fig. 8c–e, the OA group showed significantly higher levels of pro-inflammatory factors compared to the control group. In contrast, the levels of pro-inflammatory factors were reduced and anti-inflammatory factors were increased to different degrees after treatment with different agents. Notably, the reduction effect of pro-inflammatory factors and the elevation effect of anti-inflammatory factors were most pronounced in the cartilage tissue treated with HPP@Cu gel. The above results suggest that HPP@Cu gel can inhibit the production of pro-inflammatory factors and promote the release of anti-inflammatory factors by removing RONS from the joint cavity and promoting the conversion of synovial macrophages to M2.

### Biocompatibility assessment in vivo

Next, we evaluated the effects of HPP@Cu gel on the serum biochemistry, blood cells and major organs of rats. The serum biochemical results (Fig. 8g) showed that the concentrations of liver function indicators (AST, ALT) and kidney function indicators (BUN, Cre) in the serum of the rats treated with HPP@Cu gel were no significant differences as compared to the control group. The results of blood cell analysis (Additional file 1: Fig. S11) showed that the hematology of the HPP@Cu group was not significantly different from that of the control group. Moreover, no significant inflammatory lesions and histological damage were detected in the main organs of HPP@Cu group rats (Additional file 1: Fig. S12). The above results indicated that HPP@Cu gel exhibited good biocompatibility in vivo.

It has been reported that ultra-tiny Cu NPs at a dose of 4 ug/kg administered intravenously in vivo did not exhibit acute toxicity or long-term toxicity [28]. In addition, the World Health Organization has published an upper daily intake limit of 50 mg/kg/day for PVP. This dose is much higher than the total dose of Cu NDs (0.25 µg/kg) from a single injection in our in vivo experiments [73]. These data support the biocompatibility of HPP@Cu in vivo.

## Conclusions

In this study, we developed a novel multifunctional thermo-sensitive gel for the treatment of OA. Given the combination of almost all the advantages of P407, PRP, and Cu NDs, HPP@Cu gel has shown many advantages in OA treatment. HPP@Cu gel could exist as a gel in vivo due to the thermo-sensitive properties of P407, and slowly release Cu NDs, HA and PRP, prolonging the duration of pharmacological action. Given the efficient free radical scavenging properties of Cu NDs, HPP@Cu gel could scavenge RONS in the joint microenvironment and block the damaging effects of RONS on chondrocytes. In addition, our experiments showed that HPP@Cu gel could reverse the M1 polarization of macrophages and promote the production of M2 macrophages. OA rats treated with HPP@Cu gel showed no damage to articular cartilage and significantly lower levels of inflammatory factors. In summary, HPP@Cu gel presented a promising therapeutic modality for the treatment of OA.

## Methods

### Preparation of Cu NDs

Cu NDs was synthesized by hydrothermal reduction of precursor CuSO_4_ solution, according to former literature with some modifications.^40^ In a typical preparation process, anhydrous copper sulfate (0.53 mmol) was pre-dispersed in 50 ml of deionized water. Next, the above solution was added dropwise into 50 mL L-ascorbic acid solution (10 mmol) containing PVP (100 mg). The solution was kept at 80 ℃ overnight with constant stirring. After the reaction, the product was centrifuged (625×*g*, 15 min), and then the supernatant was dialyzed with water (Mw cutoff: 3500 Da) with a frequent exchange for 3 days. Cu NDs was centrifuged (11,962×*g*, 15 min) and then was dried. The obtained brown solid was used for further research.

### Preparation of HPP@Cu thermo-sensitive hydrogel

0.2% HA and thermo-sensitive materials P407 (18%) were mixed and then swollen at 4 °C for 48 h to prepare HP gel. Subsequently, the HP gel was homogeneously mixed with PRP lyophilized powder (20%) to obtain HPP gel. The previously prepared Cu NDs was dispersed in HPP gel, and then the dispersion was made uniform by a vortex machine to obtain HPP@Cu gel.

### Morphological characteristics, particle dimensions, zeta potentials, and particle stability of Cu NDs

Images of Cu NDs were acquired by TEM (JSM-6490LA, Japanese). In brief, Cu NDs dispersed in deionized water were added dropwise to an ultrathin copper mesh (300 mesh). Subsequently, the copper mesh was dried with an infrared lamp and then tested on the machine. The mean hydrodynamic diameters and zeta potentials of Cu NDs were measured with a Malvern Zetasizer laser particle size analyzer (Nano ZS90, UK). The stability of Cu NDs was assessed by monitoring the particle size variation.

### Characterization of the chemical composition of Cu NDs by XRD, FTIR, UV

Crystal structure and phase composition of Cu NDs were detected by XRD (Bruker nano8advance, Germany). FTIR spectrograms were obtained by the potassium bromide compression method using infrared spectroscopy (Nicolet iS50, Thermo Fisher Scientific). UV spectra were obtained by scanning the deionized water-dispersed Cu NDs using a UV spectrometer (UV2600, SHIMADU Corporation of Japan). The concentration of Cu NDs used in the following studies refers to the concentration of elemental copper determined by ICP-MS (Agilent7700, USA).

### H_2_O_2_ scavenging activity of Cu NDs

The scavenging ability of H_2_O_2_ was assessed by a Hydrogen Peroxide assay kit. The stable yellow complex is formed by the reaction of H_2_O_2_ with molybdate, which shows a characteristic absorption peak at 405 nm. Consequently, the concentration of the remaining H_2_O_2_ can be determined via monitoring the absorption at 405 nm of reaction products. In detail, various concentrations of Cu NDs were incubated with 20 mM H_2_O_2_ at 37 °C for 2 h, respectively. The remaining H_2_O_2_ was determined according to manufacturer’s instructions and H_2_O_2_ scavenging capacity was calculated.

### ·OH scavenging activity of Cu NDs

TMB method was employed to detect ·OH scavenging activity. The ·OH is derived from Fe^2+^-H_2_O_2_ mediated Fenton reaction, which oxidizes TMB to an oxidation product (oxTMB) with an absorbance peak in 652 nm. Firstly, the ·OH working agent was procured by dissolving 250 µM TMB, 1 mM 7H_2_O·FeSO_4_, and 30 mM H_2_O_2_ in 50 mL of NaAc-HAc buffer solution (pH 4.5, 0.5 M). Then various concentrations of Cu NDs were added to the above mixture and the reaction was carried out in the dark for 10 min. The absorbance value at 652 nm was monitored and the clearance of ·OH was calculated.

### ·O_2_^-^ scavenging activity of Cu NDs

The scavenging activity of Cu NDs on ·O_2_^-^ was assayed by Superoxide Anion Assay Kit. Briefly, the radical test solution of ·O_2_^-^ was prepared according to the provided protocol, and subsequently reacted with various concentrations of Cu NDs at 37℃ for 10 min. The absorbance at 550 nm was observed after co-incubation and the scavenging efficiency was calculated.

### PTIO radical scavenging activity of Cu NDs

PTIO·, a common oxygen radical, can be removed and discolored by antioxidants. PTIO was dissolved and diluted by deionized water. Then the PTIO test buffer was added with varying concentrations of Cu NDs and reacted for 2 h at 37 °C away from light. Afterward, the absorbance peak in 557 nm was recorded and the PTIO· eliminating capacity was calculated.

### NO· scavenging activity of Cu NDs

The NO· scavenging ability of Cu NDs was assayed by Griess reagent color development method. Sodium nitroprusside was selected as a donor of NO·. In brief, aqueous sodium nitroprusside solution was added with various concentrations of Cu NDs solution, and the reaction was carried out at 37 °C for 2 h sheltered from light, followed by the addition of Griess reagent for chromogenic development for 30 min. The absorbance at 596 nm was assayed and the clearance was counted.

### ABTS^+^ radical scavenging activity of Cu NDs

Scavenging ability of Cu NDs on free radicals was also weighed by the ABTS cation radical decolorization assay which was reported by Ni et al. [74]. ABTS^+^ radical working medium was obtained by mixing 7.4 mM ABTS diammonium salt solution with 2.6 mM potassium persulfate solution in an aqueous solution and leaving it for 12 h without light. The ABTS^+^ radical working solution was diluted with PBS (0.01 M, pH 7.4), and the Cu NDs solution was added at varying concentrations and reacted for 2 h under low light. The percentage of ABTS^+^ radical scavenged was calculated by measuring the absorbance value at 734 nm.

### DPPH radical scavenging activity of Cu NDs

DPPH radical test solution with a suitable absorbance peak at 519 nm was prepared by dissolving DPPH with ethanol, and used immediately. DPPH free radical test solution was added with Cu NDs solution of different concentrations, and the reaction was kept away from light for 30 min. After that, measured at 519 nm absorbance value, and calculated the clearance.

### Release behavior of Cu NDs, HA, and PRP-derived TGF-β1 from HPP@Cu gel

3 g of HPP@Cu gel was placed in a centrifuge tube and gelated at 37 ℃. 5 mL of pre-warmed PBS was slowly added to the gel. Then the gel was placed in a constant temperature shaker at 37 ℃ for in vitro release experiments. 1 mL of supernatant was taken at predetermined time points and the same volume of fresh medium was replenished in the centrifuge tube. The concentration of Cu in the release medium was determined by ICP-MS, and the cumulative release rate of Cu NDs was calculated. The concentration of HA in the release medium was detected by the hexadecyl trimethyl ammonium bromide (CTAB) turbidimetric method [48]. In detail, 1 mL of release medium was added to 2 mL of CTAB solution (25 mg/mL). Then the reaction was shaken for 2 min to make the reaction adequate. UV absorption was measured at 400 nm after the reaction solution was allowed to stand for 8 min. The concentration of PRP-derived TGF-β1 in the extracted medium was detected by the Rat TGF-β1 ELISA Kit. The concentration of TGF-β1 was measured according to the instructions of the kit and the cumulative release rate of PRP-derived TGF-β1 was calculated.

### Preparation of HPP@Cu gel extracts for culture medium conditions in vitro

According to ISO 10993-1 standard, P407, HPP gel, and HP gel were added to 24-well plates, respectively. After the gel was formed in the incubator at 37 °C, complete medium was added and incubated for 48 h. The supernatant was taken as the hydrogel extract. The HPP gel extract was added with Cu NDs to get HPP@Cu gel extract (Cu NDs at a concentration of 50 ng/g). HPP@Cu gel extracts were for further in vitro studies.

### Cell viability assay

Chondrocytes, L929, and RAW264.7 cells were seeded in 96-well plates respectively, and exposed to samples for 24 h. 100 µL of MTT solution was added to each well for 4 h. Subsequently, 150 µL of DMSO was added to each well for 10 min. The optical density (OD) at 492 nm was measured with a microplate reader (Infinite M200 Pro, Swiss) and the survival rate of each cell was calculated.

### Cell proliferation of HPP@Cu gel

Chondrocytes were seeded in 6-well plates and cultured with different concentrations of PRP in HPP@Cu gel. After 24 h, 48 h, and 72 h, the cells were stained with Calcein-AM/PI double staining kit (Solebro, China) and observed by inverted fluorescence microscopy.

### ROS scavenging activity assessment in vitro

Intracellular ROS scavenging activity of HPP@Cu gel was observed by confocal laser scanning microscope (CLSM, Nikon, Japan) and flow cytometer (Beckman, USA). ROS levels were detected by Reactive Oxygen Species Assay Kit (Shanghai yeasen Technology Co.). Briefly, chondrocytes and RAW264.7 cells were seeded, respectively. After incubating overnight, chondrocytes were stimulated with IL-1β (10 ng/mL) for 24 h and macrophages were activated with LPS (10 µg/mL) for 24 h to increase oxidative stress in cells. Then, cells were incubated with HPP@Cu gel for 4 h. Subsequently, DCFH-DA reactive oxygen probe was added for 30 min at 37 °C under sheltered conditions. For quantitative analysis, the cells were collected and detected by flow cytometry. To obtain fluorescent images, the nuclei of the cells loaded with the probe were stained with 4’, 6-diamidino-2-phenylindole (DAPI) for 10 min. Then, the fluorescence images of the cells were observed using a CLSM. The mean fluorescence intensity of images from three independent trials was measured by ImageJ software.

### RNS scavenging activity assessment in vitro

Intracellular RNS levels were detected by Reactive Nitrogen Assay Kit (BestBio, China). Chondrocytes or RAW264.7 cells were seeded, respectively. After incubating overnight, chondrocytes were stimulated with IL-1β (10 ng/mL) and macrophages were activated with LPS (10 µg/mL) for 24 h, then cells were incubated with HPP@Cu gel for 4 h. Subsequently, BBoxiProbe® O52 probe was added for 30 min under sheltered conditions. Then the cells were collected and detected by flow cytometry. The nuclei were stained with DAPI after probe loading. Finally, the fluorescence images were observed. The mean fluorescence intensity of images from three independent trials was measured by ImageJ software.

### Protection of chondrocytes by Cu NDs

To verify the protective effect of Cu NDs on chondrocytes, H_2_O_2_ was used to stimulate chondrocytes pretreated with Cu NDs and then the survival rate examined by standard MTT assay. Briefly, Chondrocytes were pretreated with Cu NDs for 4 h and then stimulated with 250 µM H_2_O_2_ for 24 h. Then, MTT solution was used to detect the cell viability. Moreover, the ratio of apoptotic and necrotic cells was detected by Annexin V-FITC apoptosis detection kit (Solarbio, China). Briefly, chondrocytes subjected to different treatments were collected. Then, Annexin V-FITC and PI were added for 15 min. Finally, cells were washed three times, and detected by flow cytometry.

### Polarization transition assessment of macrophages

The ability of HPP@Cu gel to reprogram the macrophage phenotype was observed by immunofluorescence staining for markers of M1 and M2. RAW264.7 was seeded in confocal culture dishes and incubated overnight, then LPS (10 µg/mL) was added to induce RAW264.7 polarization to M1 for 48 h. After polarization, the cells were treated with HPP@Cu gel for 24 h. Thereafter, the cells were fixed and blocked. After incubated with the primary antibody (CD206 and iNOS) overnight, the secondary antibody was incubated at 37 °C for 90 min. Then, the cells were washed 3 times and the fluorescent images were observed with a CLSM. Furthermore, the ability of HPP@Cu gel to reprogram the macrophage phenotype was further validated by flow cytometry assay of the percentage of M2. In brief, RAW264.7 was inoculated in 6-well plates for 12 h. Then LPS (10 µg/mL) was added to induce RAW264.7 polarization to M1 for 48 h. After that, RAW264.7 was incubated with HPP@Cu gel for 24 h. Cells were prepared suspensions, and incubated with PE-labeled CD206 antibody for 30 min. Finally, the percentage of M2 was detected by flow cytometry. Each trial was independently repeated in triplicate, and the mean fluorescence intensity was measured by ImageJ software.

### Animals

Forty-two 6-week-old female SD rats were provided by the Animal Center of Qingdao University. The study in vivo was performed under protocols approved by the Animal Management Rules of the Ministry of Health of the People’s Republic of China (document no. 55, 2001) and the examination and approval of the Experimental Animal Welfare Ethics Committee of Qingdao University (ethical approval number: 20210909SD4020211101072). The euthanasia of rats was performed by sodium pentobarbital overdose according to the ARRIVE guidelines (2020).

### Establishment of OA model induced by MIA and therapy in vivo

A rat OA model was established using joint cavity injection of MIA. Except for the control group, the rats were anesthetized and injected with 50 µL MIA (40 mg/mL) via the knee joint cavity of the right hind limb, and the treatment was started after 1 week. The rats were randomly divided into 7 groups. Control group (no treatment), OA group (saline, 50 µL), HPP@Cu group (HPP@Cu gel, 50 µL, the dosage of Cu NDs was 0.25 µg/kg), HPP group (HPP, 50 µL), HP group (HP, 50 µL), Dex group (Dexamethasone, 1 mg/kg).

### The degradation experiment of HPP@Cu gel in vivo

The healthy rats were injected by articular cavity with 50 µL of HPP@Cu gel (the dosage of Cu NDs was 0.25 µg/kg). To observe the degradation time of HPP@Cu gel, rats were dissected and the joint cavity was exposed after 1 h, 3 day and 5 day of injection, respectively. The entire joint was homogenized with saline and the concentration of the joint homogenate retained Cu NDs of the joint was measured by ICP-MS. For clearer visualization of the gel attached to the joint surface, intra-articular injections were performed using HPP@Cu gel colored with the synthetic pigment Brilliant blue. In detail, 5 µg of Brilliant blue was added to 10 g of HPP@Cu gel, and then overnight at 4 °C.

### Biocompatibility evaluation in vivo

To evaluate the biosafety of HPP@Cu gel in vivo, blood was collected in HPP@Cu group and control group after treatment for serum biochemical assays and routine blood tests. The serum biochemical assays included liver function indicators and renal function indicators. To evaluate the systemic toxicity in vivo of the formulation, the major organs were collected for H&E staining and histological analysis.

### ROS and RNS detection of articular cartilage

Intra-articular RONS scavenging activity was detected by the Tissue ROS Assay Kit (BestBio, China) and the Tissue RNS Assay Kit (BestBio, China). In brief, fresh knee cartilage tissue was taken and homogenized with buffer. Supernatant was collected by centrifuging the homogenate. Then BBcell Probe® O13 ROS probe or BBcell Probe® O52 RNS probe was added respectively for 30 min in darkness. Finally, the level of RONS was detected by a microplate reader.

### Pathological histological evaluation

The right joint tissues of rats were fixed for 48 h, decalcified for 4 weeks, and then paraffin-embedded and sectioned. The joints were subjected to staining with H&E and saffron O-solid green. Changes of cartilage tissue structure and proteoglycan were observed and cartilage degeneration was evaluated by the OARSI scoring System.

### Statistical analysis

Statistical analysis of all data was presented as mean ± standard deviation (SD). Significant differences among groups were determined by one-way analysis of variance (ANOVA) and two-tailed Student’s *t*-test. *P* < 0.05 was considered statistically significant. Superscript symbol “*” indicates comparisons with the first group, and superscript symbol “#” indicates comparisons with the second group. Comparisons between the third group and the fourth group and the fifth group and the fourth group were calculated and marked with the superscript symbol “&”. Superscript symbols “*”, “#”, and “&” indicate *p* < 0.05. Superscript symbols “**”, “##”, and “&&” indicate p < 0.01. NS means not significant.

## Supplementary Information

**Additional file 1.** **Figure S1.** The stability of Cu NDs in different media. **Figure S2.** Cu NDs scavenged free radicals in a time-dependent manner. **Figure S3.** (a) Gelation temperature of different concentrations (15%-25%) of P407. (b) Gelation time of different concentrations (15%-25%) of P407. (c) Gelation temperature of P407 (18%) introduced with different concentrations (0.10%-0.25%) of HA. (d) Gelation time of 18% P407 with the introduction of different concentrations (0.10%-0.25%) of HA. **Figure S4.** Dissolution rate curve of HPP@Cu gel incubated with PBS at 37℃. **Figure S5.** Cumulative release profile of HA from HPP@Cu gel. **Figure S6.** Cumulative release profile of PRP-derived TGF-β1 from HPP@Cu gel. **Figure S7.** RONS scavenging activity of HPP@Cu at different concentrations. **Figure S8.** RONS scavenging capacity of HPP@Cu by flow cytometric analysis. **Figure S9.** The degradation time of Bright blue colored HPP@Cu hydrogel was studied in vivo after joint cavity injection. **Figure S10.** The content of Cu NDs in rat joints was measured by ICP-MS after 1 h, 3 D and 5 D of HPP@Cu gel injection, respectively. **Figure S11.** Values of blood parameters in the normal rat (control group), and rat treated with HPP@Cu gel. **Figure S12. **System toxicity assessment of HPP@Cu gel in vivo. H&E stained images of major organs (heart, liver, spleen, lungs, and kidneys) after treatment.

## Data Availability

The datasets used and/or analyzed during the current study are available from the corresponding author on reasonable request.

## Abbreviations

OA: Osteoarthritis
P407: Poloxamer 407
HA: Hyaluronic acid
Cu NDs: Copper nanodots
PRP: Platelet-rich plasma
RONS: Reactive oxygen and nitrogen species
NSAIDs: Non-steroidal anti-inflammatory drugs
IA: Intra-articular
ROS: Reactive oxygen species
RNS: Reactive nitrogen species
Cu: Copper
TCA: Tricarboxylic acid cycle
Cu NDs: Copper nanodots
TEM: Transmission electron microscopy
DLS: Dynamic light scattering
FBS: Fetal bovine serum
XRD: X-ray diffraction
PVP: Polyvinyl pyrrolidone
FTIR: Fourier transform infrared
UV-*vis*: Ultraviolet-visible
·O_2_^-^: Superoxide anion
PDGF: Platelet-derived growth factor
TGF-β: Transforming growth factor-β
IGF: Insulin-like growth factor
DCFH-DA: 2′-7′-Dichlorodihydrofluorescein diacetate
LPS: Lipopolysaccharide
IL-4: Interleukin-4
MIA: Sodium iodoacetate
Dex: Dexamethasone
H&E: Hematoxylin and eosin
OARSI: Osteoarthritis Research Society International
OD: Optical density
CLSM: Confocal laser scanning microscope
DAPI: 4’,6-Diamidino-2-phenylindole
CTAB: Hexadecyl trimethyl ammonium bromide
