# Supplementary material for: Multifunctional thermo-sensitive hydrogel for modulating the microenvironment in Osteoarthritis by polarizing macrophages and scavenging RONS
Source: J Nanobiotechnology. 2022 May 7;20:221. doi: 10.1186/s12951-022-01422-9 (PMC9077879; doi:10.1186/s12951-022-01422-9)
Supplement: Supplementary file 1 — Additional file 1. Figure S1. The stability of Cu NDs in different media. Figure S2. Cu NDs scavenged free radicals in a time-dependent manner. Figure S3. (a) Gelation temperature of different concentrations (15%-25%) of P407. (b) Gelation time of different concentrations (15%-25%) of P407. (c) Gelation temperature of P407 (18%) introduced with different concentrations (0.10%-0.25%) of HA. (d) Gelation time of 18% P407 with the introduction of different concentrations (0.10%-0.25%) of HA. Figure S4. Dissolution rate curve of HPP@Cu gel incubated with PBS at 37℃. Figure S5. Cumulative release profile of HA from HPP@Cu gel. Figure S6. Cumulative release profile of PRP-derived TGF-β1 from HPP@Cu gel. Figure S7. RONS scavenging activity of HPP@Cu at different concentrations. Figure S8. RONS scavenging capacity of HPP@Cu by flow cytometric analysis. Figure S9. The degradation time of Bright blue colored HPP@Cu hydrogel was studied in vivo after joint cavity injection. Figure S10. The content of Cu NDs in rat joints was measured by ICP-MS after 1 h, 3 D and 5 D of HPP@Cu gel injection, respectively. Figure S11. Values of blood parameters in the normal rat (control group), and rat treated with HPP@Cu gel. Figure S12. System toxicity assessment of HPP@Cu gel in vivo. H&E stained images of major organs (heart, liver, spleen, lungs, and kidneys) after treatment. [file 12951_2022_1422_MOESM1_ESM.docx]

***Additional file 1***

**Multifunctional thermo-sensitive hydrogel for modulating the microenvironment in Osteoarthritis by polarizing macrophages and scavenging RONS**

**1. EXPERIMENTAL SECTION**

## 1.1 Materials

Plasdone K-29/32 (PVP) was purchased from ISP Corporation (Wayne,N.J., USA). Cupric sulfate anhydrous (CuSO_4_), L-Ascorbic acid, ABTS, TMB, DPPH, sodium nitroprusside dehydrate, Brilliant blue, and Dexamethasone (Dex) were purchased from Maclean Biochemical Technology Co., Ltd. (Shanghai, China). 7H_2_O∙FeSO_4_, PTIO, sulfanilic acid, hexadecyl trimethyl ammonium bromide (CTAB) and N-(1-naphthyl) ethylenediamine dihydrochloride were purchased from Aladdin Industrial Corporation (Shanghai, China). Reactive Oxygen Species Assay Kit was purchased from Yeasen BioTechnologies Co. Ltd. (Shanghai, China). Reactive Nitrogen Species Assay Kit, Tissue Reactive Oxygen Species Assay Kit and Tissue Nitrogen Oxygen Species Assay Kit were purchased from Beibo Biological Technology Co., Ltd. (Shanghai, China). CD206 and iNOS antibodies were purchased from Proteintech tech (Wuhan, China). MTT, interleukin-4 (IL-4), Rat TGF-β1 ELISA kit, and DAPI were purchased from Solarbio Corp (Beijing, China). Fetal bovine serum (FBS), trypsin–EDTA, phosphate buffered saline (PBS) and Dulbecco’s modified Eagle’s medium (DMEM) were obtained from Promise Life Technology Co., Ltd. (Wuhan, China). Hyaluronic acid（HA, M_W_ 1000k-1500k）and lipopolysaccharides (LPS) were obtained from Yuanye Biotechnology Co., Ltd (Shanghai, China). Hydrogen Peroxide assay kit and Superoxide Anion Assay Kit were purchased from Jiancheng Bioengineering Institute (Nanjing, China). Female SD rats (6 weeks, 180-200 g) were purchased from SPF Biotechnology Co., Ltd. (Beijing, China).

## 1.2 Extraction and activation of platelet-rich plasma（PRP）

PRP was extracted and activated followed by a previously reported method^1^. Anticoagulant whole blood was centrifuged (5000 ×g) to discard the bottom layer of blood cells. The supernatant was subsequently centrifuged (5000 ×g) again to separate the bottom buffer layer. PRP was acquired by suspension of the bottom white turbid layer. Subsequently, the extracted PRP was mixed 4:1 with a 10% wt CaCl_2_ solution containing 1000 U/mL thrombin to activate the PRP. Activated PRP was lyophilized to maintain growth factor expression.

## 1.3 Preparation of HPP@Cu for culture medium conditions

According to ISO 10993-1 standard, P407, HPP gel, and HP gel were added to 24-well plates, respectively. After the gel was formed in the incubator at 37°C, complete medium was added and incubated for 48 h. The supernatant was taken as the hydrogel extract. The HPP gel extract was added with Cu NDs to get HPP@Cu gel extract. HPP@Cu gel extracts were for further *in vitro* studies.

## 1.4 Rheological characterization of HPP@Cu

The gelation time of the composite thermo-sensitive hydrogels was determined by inversion of the test tubes. Various concentrations of P407 (15-25 wt%) and 18 wt% of P407 containing various concentrations of HA (0.1%-0.25 wt%) were separately transferred to glass tubes at 4°C and placed in a constant temperature water bath at 37°C. The time until the gel stopped flowing when the tubes were inverted was the gelation time.

The phase transition temperature and viscosity of the sol-gel of the sample were measured by a rotational rheometer (Malvern Kinexus lab^+^, UK). The storage modulus (*G′*), loss modulus (*G″*) and complex viscosity (*η**) of the samples were detected at a constant shear stress of 1 Pa and a frequency of 1 Hz. The samples in solution state were transferred to a parallel plate at 4°C and warmed up at a rate of 1°C/min in the range of 15°C to 45°C, and the curves of *G'* and *G"* with temperature were recorded. The temperature at the intersection of *G'* and *G"* is the gelation temperature of the sample. The same shear stress and temperature parameters were set by the rheometer to detect the profile of the shear viscosity (*η*) of the sample with temperature.

## 1.5 Dissolution performance of HPP@Cu gel

First, 3 g of HPP@Cu gel was placed at 37°C to form a gel and then 5 mL PBS preheated at 37°C was slowly added. The experiment was performed at a constant temperature of 37°C and an oscillation rate of 100 r/min. The height of the hydrogel was measured at predetermined time intervals (days 1, 3, 5, 8, 12, 15, and 18), respectively. The dissolution rate (DR) of the hydrogel was calculated according to the equation (1):

DR(%) = ((H_0_-H_T_)/H_0_)×100 (1)

Where H_0_ is the initial height of the hydrogel, and H_T_ is the height of the hydrogel at the measurement time point, respectively.

## 1.6 Isolation and culture of chondrocytes

Primary chondrocytes were extracted from the knee cartilage of 1-week-old SD suckling rats, as described previously. SD rats were anesthetized and executed, followed by immersion in 75% alcohol for 10 min. Thigh knee cartilage was detached under aseptic conditions, removing as much fascia, muscle, and connective tissue as possible. The separated cartilage was rinsed in PBS, chopped and digested with trypsin-EDTA for 30 min. After that, the cartilage fragments were digested by vibrating with 0.2% collagenase II (Solarbio, USA) at 37°C for 4h. Single cells were obtained by filtering the supernatant through a cell sieve. The 3rd-5th generation cells were used for further experiments.

## 1.7 Validation of phenotypic polarization of macrophages

CLSM and flow cytometry analysis were used to jointly verify that LPS induces macrophage polarization to M1 phenotype and that IL-4 promotes macrophage polarization to M2 phenotype.

RAW264.7 in the logarithmic growth phase was seeded in confocal culture dishes and incubating for 12 h. Then LPS (10 μg/mL) or IL-4 (50 ng/mL) was added to induce RAW264.7 polarization for 48 h, respectively. After polarization, cells were fixed and blocked. Then the supernatant was discarded, primary antibody was added and incubated overnight. After washing off the primary antibody, the secondary antibody was incubated at 37°C for 90 min. Then, the cells were washed 3 times and the fluorescent images were observed with a CLSM.

RAW264.7 at logarithmic growth phase was inoculated in 6-well plates for 12 h. Then LPS (10 μg/mL) or IL-4 (50 ng/mL) was added to induce RAW264.7 polarization, respectively. After 48 h of polarization, cells were washed, digested and centrifuged to prepare cell suspensions, and incubated with FITC-labeled CD206 antibody for 30 min. Finally, the cells were resuspended with PBS, and detected using flow cytometry.

## 1.8 Statistical analysis

Statistical analysis of all data was presented as mean ± standard deviation (SD). Significant differences among groups were determined by one-way analysis of variance (ANOVA) and two-tailed Student's *t*-test. *P* < 0.05 was considered statistically significant. Superscript symbol “*” indicates comparisons with the first group, and superscript symbol “#” indicates comparisons with the second group. Comparisons between the third group and the fourth group and the fifth group and the fourth group were calculated and marked with the superscript symbol “&”. Superscript symbols “*”, “#”, and “&” indicate *p* < 0.05. Superscript symbols “**”, “##”, and “&&” indicate p < 0.01. NS means not significant.

**2. SUPPORTING FIGURES**

**
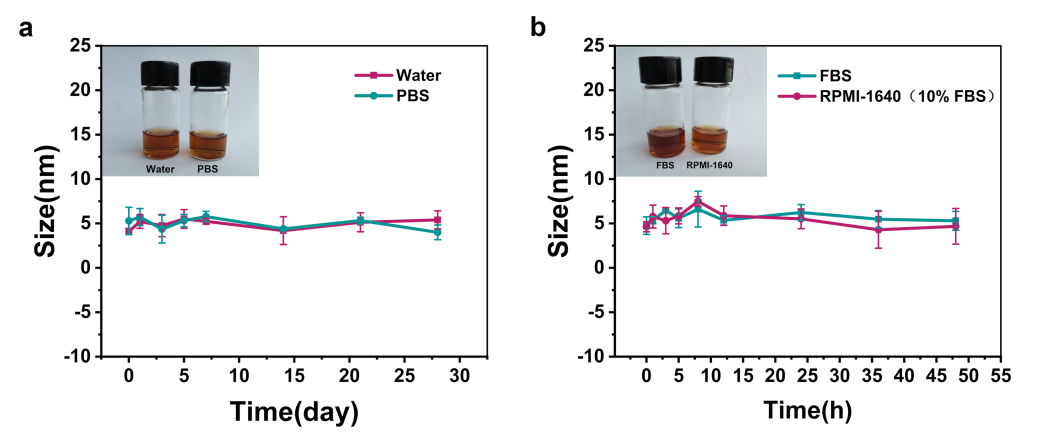
**

**Figure S1. The stability of Cu NDs in different media. (a) The mean diameter of Cu NDs in water and PBS during 28 days, respectively. (b) The mean diameter of Cu NDs in FBS and complete medium during 48 h, respectively. Date were presented as mean ± SD (n=3). The inserted images are of the Cu NDs dispersed in different media.**

**
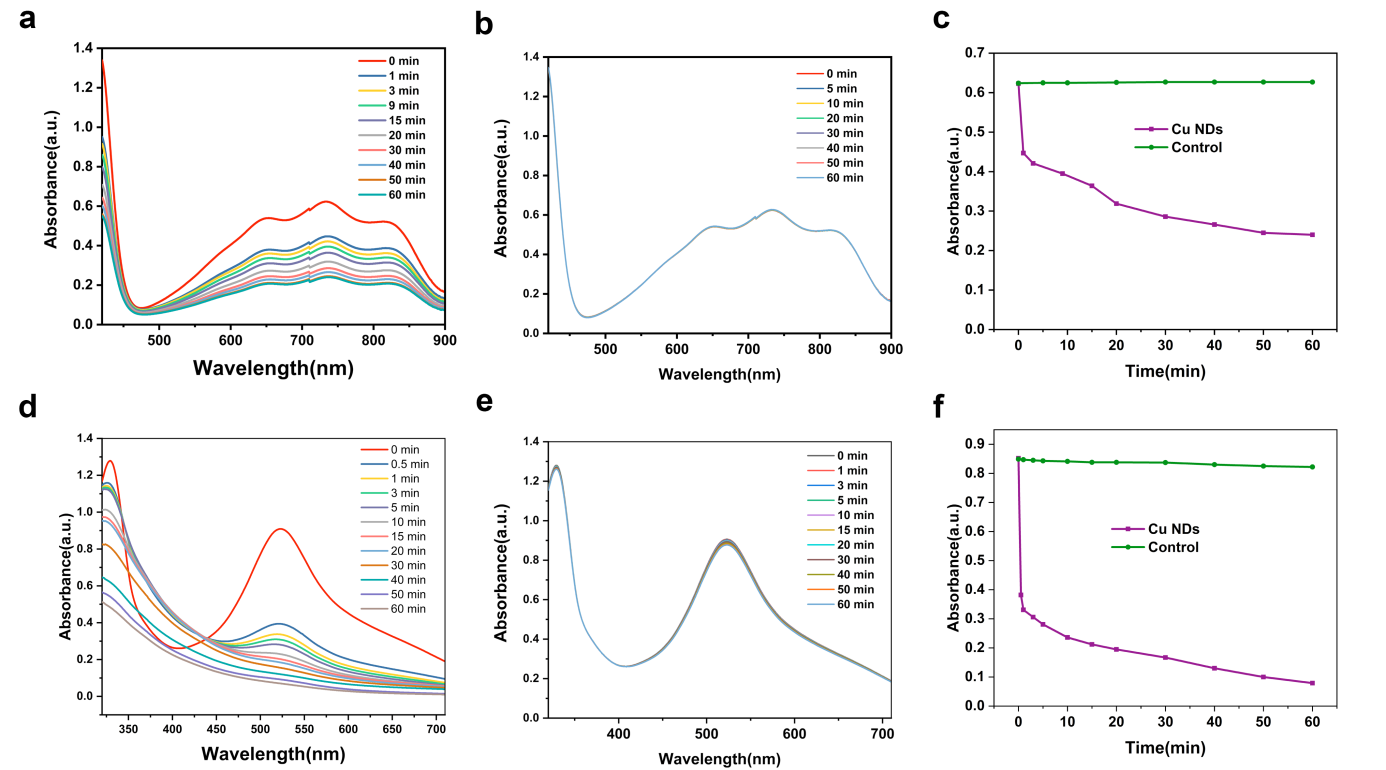
**

**Figure S2.** **Cu NDs scavenged free radicals in a time-dependent manner. (a)** **Absorption spectra of ABTS^+^ testing solution treated with Cu NDs (5 ng/mL) during 60 min. (b) Absorption spectra of ABTS^+^ testing solution without Cu NDs during 60 min. (c) Absorbance value at 734 nm of ABTS^+^ testing solution after mixed with or without Cu NDs. (d)** **Absorption spectra of DPPH solution treated with Cu NDs (3 ng/mL) during 60 min. (e)** **Absorption spectra of DPPH solution without Cu NDs during 60 min. (f)Absorbance value at 509 nm of DPPH solution after mixed with or without Cu NDs.**

**
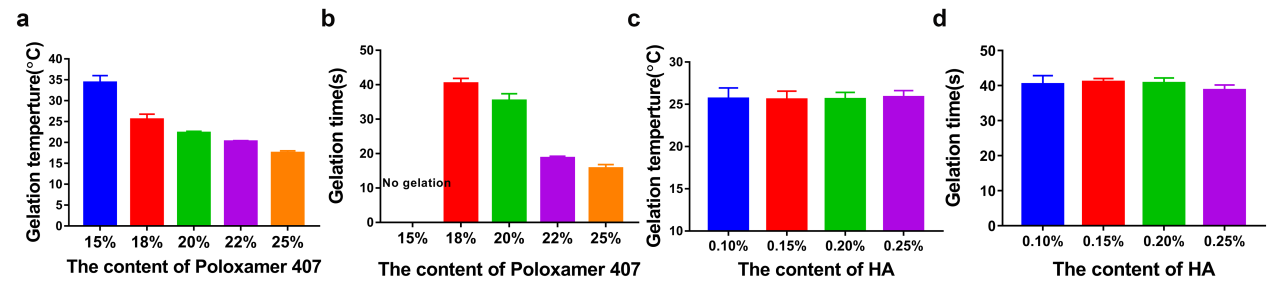
**

**Figure S3. (a) Gelation temperature of different concentrations (15%-25%) of P407. (b) Gelation time of different concentrations (15%-25%) of P407. (c) Gelation temperature of P407 (18%) introduced with different concentrations (0.10%-0.25%) of HA. (d)** **Gelation time of 18% P407 with the introduction of different concentrations (0.10%-0.25%) of HA. Date were presented as mean ± SD (n=3).**

**

**

**Figure S4. Dissolution rate curve of HPP@Cu gel incubated with PBS at 37℃. Date were presented as mean ± SD (n=3).**

**

**

**Figure S5. Cumulative release profile of HA from HPP@Cu gel. Date were presented as mean ± SD (n=3).**

**

**

**Figure S6.** **Cumulative release profile of PRP-derived TGF-β1 from HPP@Cu gel. Date were presented as mean ± SD (n=3).**

**
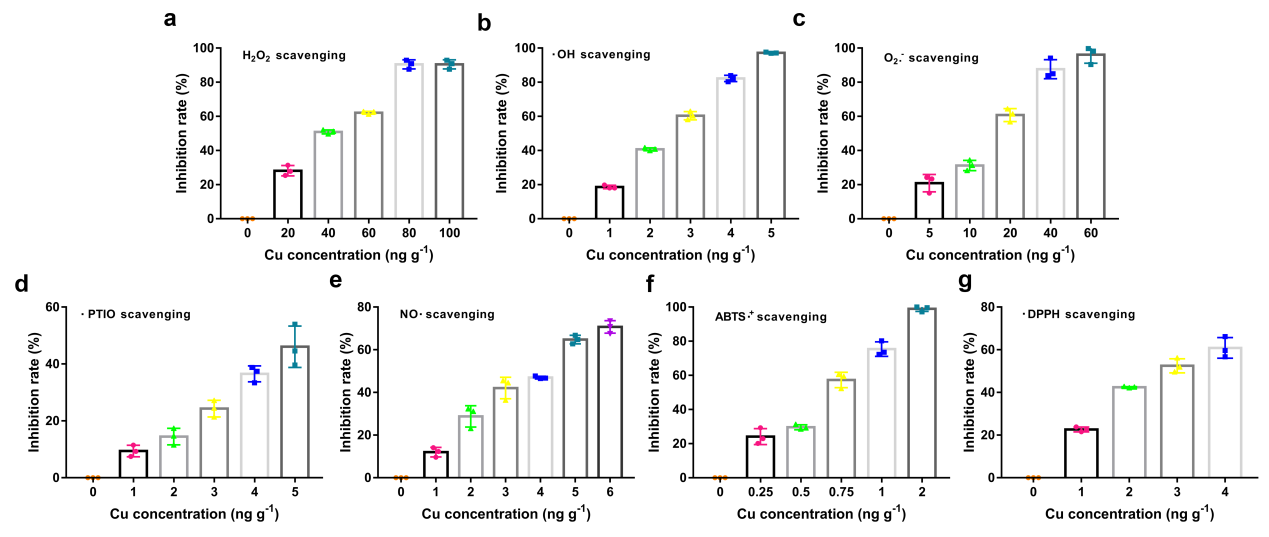
**

**Figure S7. RONS scavenging activity of HPP@Cu at different concentrations.** **(a) H_2_O_2_ scavenging activity of Cu NDs at various concentrations. (b) NO∙ scavenging activity of Cu NDs at various concentrations. (c) ∙O_2_^-^ scavenging activity of Cu NDs at various concentrations. (d) ∙PTIO scavenging activity of Cu NDs at various concentrations. (e) NO∙ scavenging activity of Cu NDs at various concentrations. (f) ABTS∙^+^ scavenging activity of Cu NDs at various concentrations. (g) ∙DPPH scavenging activity of Cu NDs at various concentrations. Date were presented as mean ± SD (n=3).**

**
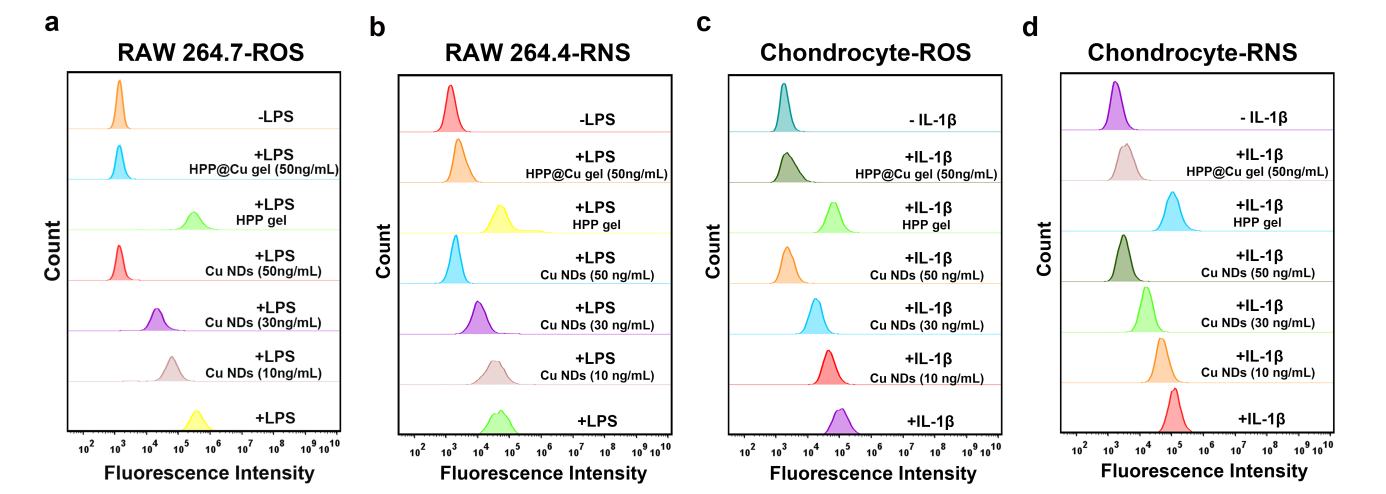
**

**Figure S8. RONS scavenging capacity of HPP@Cu by flow cytometric analysis. (a) ROS levels of RAW264.7 after different treatments. (b) RNS levels of RAW264.7 after different treatments. (c) ROS levels of chondrocyte after different treatments. (d) RNS levels of chondrocyte after different treatments.**

**
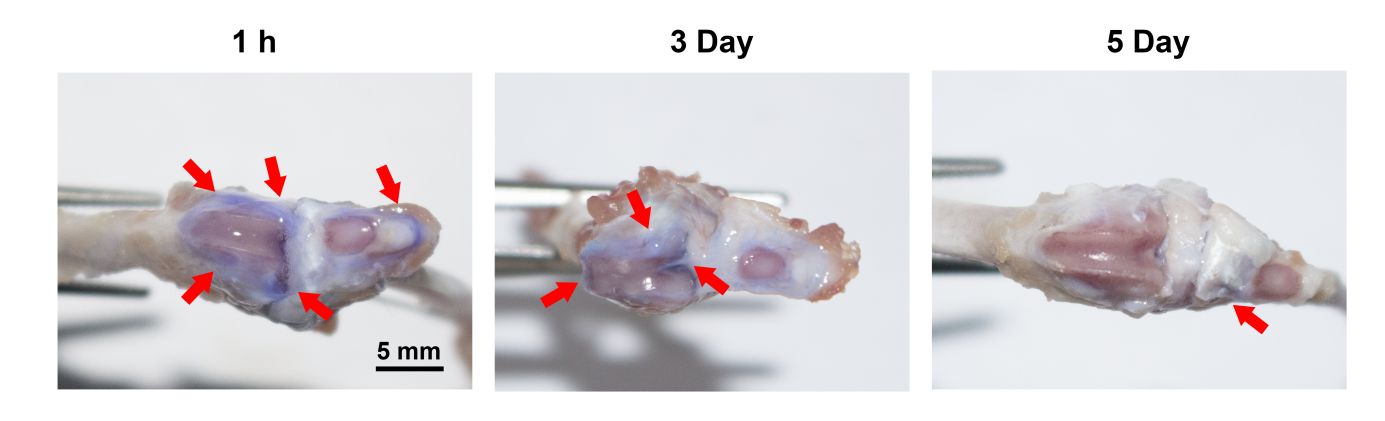
**

**Figure S9.** **The degradation time of Bright blue colored HPP@Cu hydrogel was studied *in vivo* after joint cavity injection.** **The red arrows direct the location of the gel.**

**

**

**Figure S10. The content of Cu NDs in rat joints was measured by ICP-MS after 1 h, 3 D and 5 D of HPP@Cu gel injection, respectively. Date were presented as mean ± SD (n=3).**


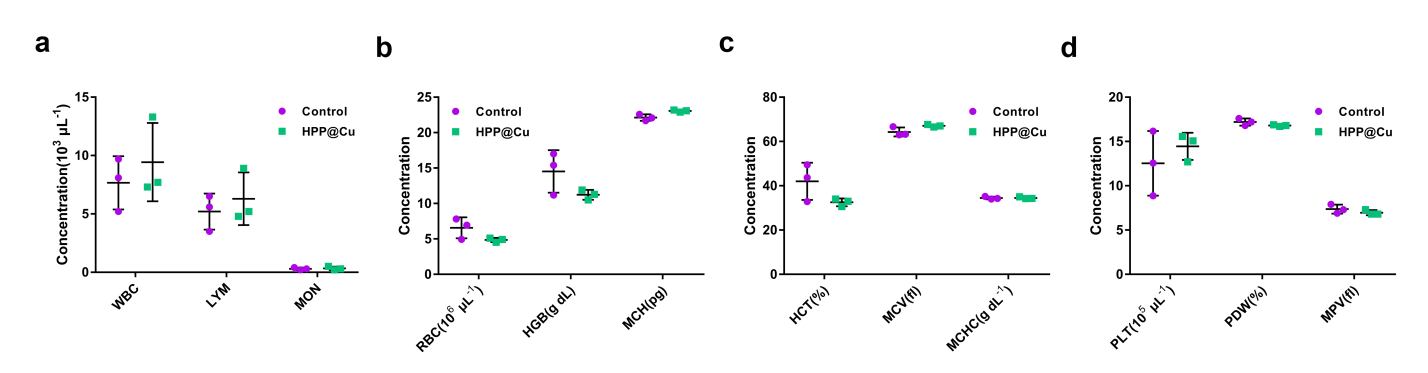


**Figure S11. Values of blood parameters in the normal rat (control group), and rat treated with HPP@Cu gel. Date were presented as mean ± SD (n=3).**

**
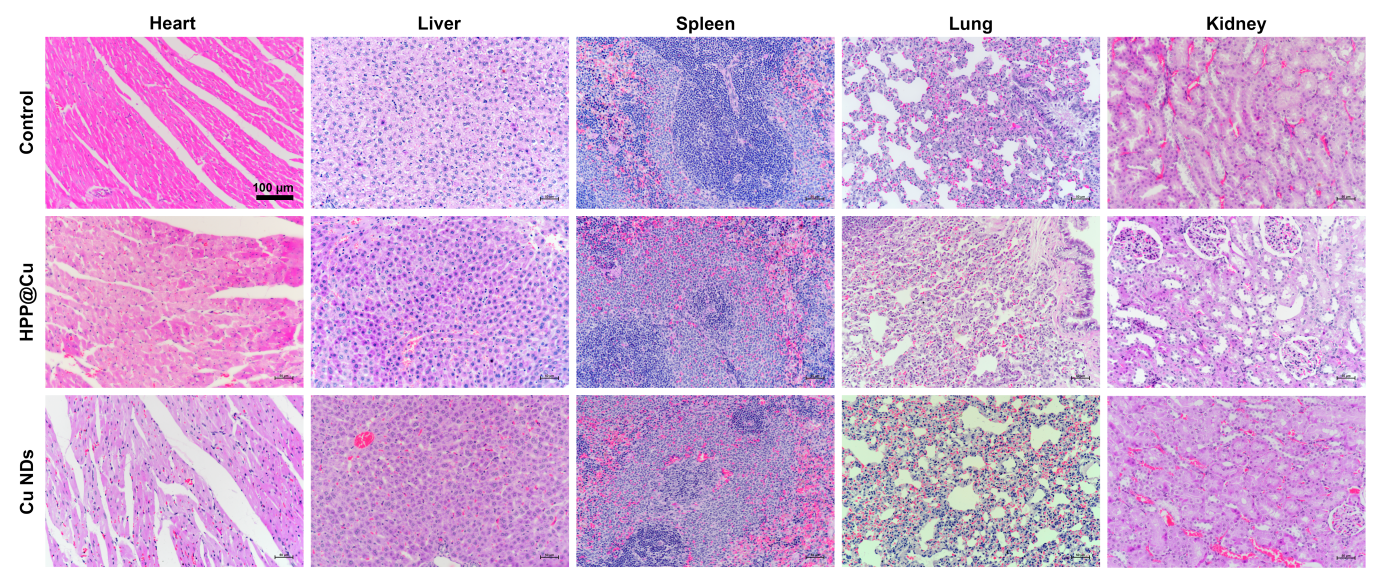
**

**Figure S12. System toxicity assessment of HPP@Cu gel *in vivo*. H&E stained images of major organs (heart, liver, spleen, lungs, and kidneys) after treatment.**

**References**

1. Lee, J.; Kim, G., Calcium-Deficient Hydroxyapatite/Collagen/Platelet-Rich Plasma Scaffold with Controlled Release Function for Hard Tissue Regeneration. *ACS Biomater Sci Eng* **2018,** *4* (1), 278-289.
